# Activation of NF-κB signaling in tissue-resident memory T cells promotes recurrent psoriasis in mice

**DOI:** 10.3389/fimmu.2025.1762269

**Published:** 2026-02-09

**Authors:** Yukang Lin, Jiaying Chen, Han Du, Yuchao Chen, Zhaolin Liu, Xuejia Li, Yongdan Li, Feifei Qiu, Lingling Wen, Siyuan Xu, Huazhen Liu

**Affiliations:** 1 State Key Laboratory of Traditional Chinese Medicine Syndrome, The Second Affiliated Hospital of Guangzhou University of Chinese Medicine, Guangzhou, China; 2 Maoming Hospital of Guangzhou University of Chinese Medicine, Maoming, China; 3 Shenzhen Traditional Chinese Medical Hospital, Shenzhen, China; 4 The Third Affiliated Hospital of Guangzhou University of Chinese Medicine, Guangzhou, China; 5 Sci-tech Industrial Park of Guangzhou University of Chinese Medicine, Guangzhou, China

**Keywords:** NF-κB signaling, recurrent psoriasis, tissue resident memory T cells, imiquimod, inflammation

## Abstract

**Background:**

Recurrence triggered by immunological memory is a critical challenge in the treatment of psoriasis. Tissue-resident memory T (Trm) cells are the primary pacemakers in recurrent psoriasiform dermatitis following stimulation and infection by previous pathogens. However, the mechanisms underlying Trm cell activation remain unclear.

**Methods:**

In this study, imiquimod-induced recurrent psoriatic mice were established to characterize the phenotypes of different Trm cell subsets. CD8^+^/CD4^+^ Tcm cell injection and NF-κB inhibitor or agonist treatment were then used to investigate the role and mechanisms of Trm cells in recurrent psoriasis. Finally, CD8^+^ T cells and keratinocyte co-culture systems were established to investigate the effects of activating NF-κB activation in Trm cells.

**Results:**

Mice displayed severe psoriatic dermatitis after repeated imiquimod treatment. The CD8^+^ Trm cells, but not CD4^+^ Trm cells, were elevated in the skin of recurrent psoriatic mice. Injection of CD8^+^ Tcm cells injection elicited more severe psoriatic symptoms than imiquimod treatment alone, indicating that CD8^+^ Trm cells are critical participants in psoriatic recurrence. Phosphorylation of NF-kB p65 (RELA), p-IKKa, p-RelB and NF-kB p100/p52 was enhanced in the skin of imiquimod-induced recurrent psoriatic mice. NF-κB inhibitor/agonist treatment significantly suppressed or restored the dermatitis severity and CD8^+^ Trm cell levels in recurrent psoriatic mice. Meanwhile, NF-κB inhibition also restored the expression of DLAT in Trm cells. Finally, NF-κB inhibitors directly suppressed Trm cell activation and inflammation of Trm cells *in vitro*.

**Conclusion:**

Together, these findings suggest that canonical and non-canonical NF-κB signaling directly activates Trm cell differentiation, inhibits cellular cuproptosis, and promotes recurrent psoriasis.

## Introduction

Psoriasis is a chronic, papulosquamous dermatitis and is associated with many other complications. Over 125 million people are affected by psoriasis worldwide (1). The primary pathogenesis of psoriasis involves aberrant activation and dysregulation of both innate and adaptive immunity. Although immunosuppressants and biological therapies display satisfactory effects in treating psoriasis (2), most of patients still suffer from the torment of recurrent psoriasis (3). Revealing the pathogenic mechanisms is critical for preventing recurrent psoriasis.

Repeated stimulation or infection by previous pathogens is the direct etiology of recurrent psoriasis (40). Memory T cell–mediated immunological memory plays a crucial role in immune responses to repeated infection and stimulation. A proportion of T cells can differentiate into effector memory T (Tem) cells, central memory T (Tcm) cells, and tissue-resident memory T (Trm) cells (4). Unlike Tem/Tcm cells that circulate between the blood and lymph nodes (LNs), Trm cells show low migration and long-term survival in peripheral tissues, such as the skin (3, 5). Although cutaneous Trm cells provide rapid immune protection against previous pathogen infections, they also trigger the pro-inflammatory responses in recurrent inflammatory dermatoses (6–8). In psoriasis, cutaneous Trm cells can persist after effective treatment and promote psoriatic recurrence by producing inflammatory cytokines, including IL-17A (9, 10). Hence, inhibiting the generation/retention of Trm cells in the skin could be a reasonable strategy to prevent psoriatic recurrence. However, the crucial mechanisms underlying activation of cutaneous Trm cells in recurrent psoriasis remain unclear.

The nuclear factor-κB (NF-κB) family is a classic protein complex that modulates both innate and adaptive immunity (11). It is essential for lymphocyte activation and survival by regulating the transcription of a large number of genes (12). Activating NF-κB activation can occur through canonical and non-canonical NF-κB signaling pathways (12). The canonical NF-κB signaling pathway activates NF-κB p50, RELA, and c-REL, whereas the non-canonical NF-κB signaling pathway activates NF-κB p52 and RELB (12). These protein complexes are bound to and inactivated by inhibitors of NF-κB (IκBs) (11). Upon activation, IκB proteins are degraded, allowing NF-κB–associated proteins to translocate into the nucleus (11). Previous studies have shown that NF-κB signaling plays a critical role in the generation and maintenance of effector and memory T cells (13–15). However, the effects of NF-κB signaling on Trm cell activation of Trm cells in psoriasis remain unknown.

In this study, a repeated imiquimod-induced recurrent psoriatic mouse model was established to identify the phenotypes of different Trm cell subsets (CD4^+^ and CD8^+^). Then, Tcm cell injection and NF-κB inhibitor or agonist treatment were used to investigate the role of CD8^+^ Trm cells and the effects of NF-κB signaling in recurrent psoriasis. Finally, we established a co-culture system consistingof keratinocytes and CD8^+^ T cells, aiming to investigate the impacts of directly triggering NF-κB activation within CD8^+^ Trm cells.

## Materials and methods

All experimental procedures involving animals were approved by the Animal Ethics Committee of Guangdong Provincial Academy of Chinese Medical Sciences.

### Reagents

Imiquimod cream was obtained from Mingxin Pharmaceutical Co., Ltd. (Sichuan, China). CU-T12–9 and NIK SMI 1 were obtained from MedChemExpress (Shanghai, China). Recombinant murine IL-15 (rIL-15) was obtained from PeproTech (Rocky Hill, USA).

### Recurrent psoriatic mice model

**Figure 1:**
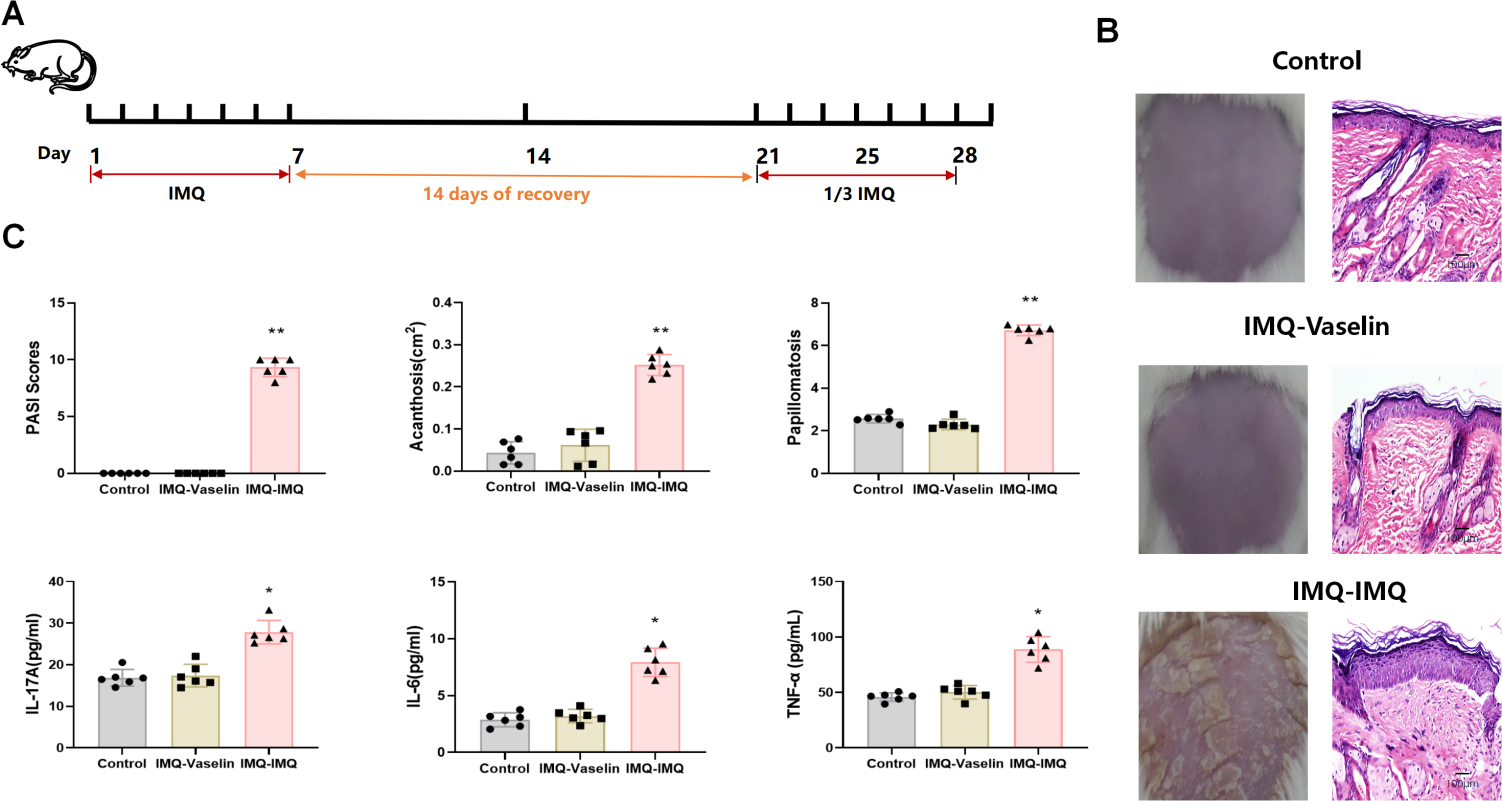
Imiquimod (IMQ)-induced recurrent psoriatic mice. Flow chart of IMQ-induced recurrent psoriasis **(A)**, representative macroscopic images and H&E-stained dorsal skin sections **(B)**, and cutaneous PASI scores, acanthosis, papillomatosis, IL-17A, IL-6, and TNF-α levels in psoriatic mice **(C)**. Values are means ± SD, n = 6 per group. *P<0.05, **P<0.01 versus control group.

A recurrent psoriatic mouse model was established according to our previous research (16). Seven-week-old male BALB/c mice were obtained from the Guangdong Medical Laboratory Animal Center (Guangzhou, China). Mice were housed under pathogen-free conditions in a temperature-controlled room with a 12-h light/dark cycle and received humane care. Mice were divided into the control, imiquimod (IMQ)–vaseline, and IMQ–IMQ groups. After shaving the back hair was shaved, all mice except those in the control group were treated with 62.5 mg/day IMQ for 7 consecutive days. After immunologic tolerance for 14 days (a treatment-free resting period following initial IMQ application), mice in the IMQ–vaseline group were received vaseline treatment for another 7 consecutive days. Mice in the IMQ–IMQ group were received 20.8 mg/day IMQ for 7 consecutive days to induce recurrent psoriasis (**Figure 1A**). The severity of psoriasis was evaluated using the Psoriasis Area and Severity Index (PASI), acanthosis, and papillomatosis. PASI was assessed based on three parameters (skin erythema, scaling, and thickness). Each independent parameter was scored as follows: 0, none; 1, slight; 2, moderate; 3, marked; and 4, very marked. To measure acanthosis, the epidermal area was outlined, and its pixel size was measured. The relative area of the epidermis was calculated using the following formula as follows: area = pixels/(horizontal resolution × vertical resolution). The papillomatosis index was typically measured as previously reported (17).

### Histology

Skin tissues were fixed in formaldehyde, paraffin-embedded, sectioned, and stained with hematoxylin and eosin.

### Biochemical assays

Skin tissues were homogenized in RIPA lysis buffer. Cutaneous IL-17A, IL-15, IL-6, and TNF-α levels were determined using commercial ELISA kits (Boster, China).

### Flow cytometric analysis

The flow cytometric analysis protocol was performed according to our previous research (16). LNs and spleens were harvested and homogenized. Cells were collected and stained with anti-CD4 PE antibody (Clone GK1.5, eBioscience, USA), anti-CD8a FITC antibody (Clone 53-6.7, eBioscience, USA), anti-CD44 eFluor™ 450 antibody (Clone IM7, eBioscience, USA), and anti-CD62L APC antibody (Clone MEL-14, eBioscience, USA). Skin tissues were digested with collagenase type I (Sigma-Aldrich, USA) and DNase (Solarbio, Shanghai, China). After erythrocyte lysis, cells were stained with anti-CD8a Alexa Fluor™ 700 antibody (Clone 53-6.7, eBioscience, USA), anti-CD103 PE antibody (Clone 2E7, eBioscience, USA), anti-CD69 PE-Cyanine7 antibody (Clone H1.2F3, eBioscience, USA), anti-CD4 Alexa Fluor™ 700 antibody (Clone GK1.5, eBioscience, USA), anti-CLA PE antibody (Clone HECA-452, BioLegend, Beijing, China), anti-CD44 eFluor™ 450 antibody (Clone IM7, eBioscience, USA), anti-CD62L APC antibody (Clone MEL-14, eBioscience, USA), and anti-DLAT antibody (MA5-53986, Thermo Fisher Scientific, USA). Cells were analyzed using a flow cytometer (NovoCyte Quanteon, Agilent, USA).

### Real-time polymerase chain reaction

Skin tissues were homogenized in TRIzol reagent (Invitrogen, Carlsbad, USA). Total RNA was isolated and synthesized into single-stranded cDNA. Finally, Quantitative real-time PCR was performed using a ViiA 7 Dx system (Applied Biosystems, Carlsbad, USA) with SYBR Premix ExTaqTM Π (Takara Bio Incorporation, Tokyo, Japan). The primer sequences are shown below:

CXCL9 Forward: CTCGGCAAATGTGAAGAAGCTGA;CXCL9 Reverse: TTCCTTGAACGACGACGACTTTGG; IL-15 Forward: TCCATCTCGTGCTACTTGTGTTTCC; IL-15 Reverse: TCCCTATGGCCCTCATTCTCACTG; CXCL10 Forward: TGCCGTCATTTTCTGCCTCATCC; CXCL10 Reverse: CACATTCTGGAGGAAGTCCTTGG; CXCR3 Forward: CAGCCCTCTACAGCCTCCTCTTC;CXCR3 Reverse: TACAGCCAGGTGGAGCAGGAAG; β-actin Forward: GGCTGTATTCCCCTCCATCG, β-actin Reverse: CCAGTTGGTAACAATGCCATGT.

### Western blot

Skin tissues were homogenized in RIPA lysis buffer as described above. Total protein was isolated and separated by SDS-PAGE. After transfer to PVDF membranes, membranes were blocked and incubated with anti-GAPDH (Cell Signaling Technology, Boston, USA), anti-phospho-NF-κB p65, (Cell Signaling Technology, Boston, USA), anti-NF-κB-p100/p52, anti-p-IKKα, anti-p-RelB, (Cell Signaling Technology, Boston, USA), anti-phospho-TRAF2, and anti-phospho-p38 MAPK antibodies (Cell Signaling Technology, Boston, USA). After incubation with the appropriate secondary antibodies, membranes were visualized using a Bio-Rad gel imaging system and analyzed with Image Lab software.

### CD8^+^/CD4^+^ Tcm cells and rIL-15 treatment

To purify CD4^+^CD44^+^CD62L^+^ cells or CD8^+^CD44^+^CD62L^+^ cells for adoptive transfer experiments, spleen cells from naïve wild-type mice were stained with anti-CD4 Abs, anti-CD8 Abs, anti-CD44 Abs and anti-CD62L Abs (BD Biosciences, USA). CD4^+^CD44^+^CD62L^+^ cells or CD8^+^CD44^+^CD62L^+^ cells were then sorted out by FACSAria III (BD Biosciences). The purity of the sorted CD4^+^ Tcm and CD8^+^ Tcm is typically >85%. 

Mice were divided into control, IMQ–IMQ, CD8^+^ Tcm, CD4^+^ Tcm, and rIL-15 groups. All mice except those in the control group were induced to develop recurrent psoriasis mice as described above. Mice in the CD8^+^ Tcm or CD4^+^ Tcm groups were injected with exogenous CD4^+^ or CD8^+^ Tcm cells (1×10^^6^) on day 21, respectively. Mice in the rIL-15 group were received recombinant murine IL-15 (*i.p.*, 0.1 μg/mouse) on days 21, 24, and 27. These Tcm cells were isolated from mice treated with IMQ on day 21.

### NF-κB inhibitor/agonist treatment

Mice were divided into control, IMQ–IMQ, NIK SMI 1, and CU-T12–9 groups. All mice except those in the control group were induced to develop recurrent psoriasis as described above. Mice in the NIK SMI 1 and CU-T12–9 groups received 20 mg/kg NIK SMI 1 (*i.p.*) or CU-T12-9 (60 mg/kg, *i.p.*) on days 21, 24, and 27, respectively.

### Cell sorting and culture

To obtain mouse keratinocytes, the skin of 4–7-day-old BALB/c pups was isolated and the tissue was incubated in a dissociation enzyme solution at 4 °C for 16–21 h. After epidermal separation, TrypLE™ Express (Gibco) was added and incubated at 37 °C with shaking for 30 min. Digestion was terminated by adding primary keratinocyte culture medium (PriMed-iCell-010, iCell, China). After filtration and centrifugation, the keratinocytes were resuspended and collected. Primary keratinocytes were cultured in primary keratinocyte medium (PriMed-iCell-010, iCell, China). To induce a psoriasis-like model *in vitro*, recombinant mouse IL-17A (10 ng/mL) was added to the culture supernatant. For the model group, prior to co-culture, the sorted CD8^+^ T cells were pre-differentiated into tissue-resident memory T cells (Trm cells) by induction with recombinant IL-15 (50 ng/mL), NIK SMI 1 and TGF-β (50 ng/mL) Subsequently, these pre-differentiated CD8+ Trm cells were further used to treat the keratinocytes in the co-culture experiment for 48 h. For the NIK SMI 1 group, the keratinocytes were further treated with CD8^+^ Trm cells which induced with recombinant IL-15, TGF-β, and NIK SMI 1 for 48 h. All cells were maintained in a humidified incubator at 37 °C with 5% CO_2_.

### Data analysis

All results are expressed as means ± standard deviation. Data were analyzed using the Kolmogorov–Smirnov test for normality. The Mann–Whitney U test was used for non-normally distributed data. Data from more than two groups were analyzed using one-way analysis of variance (ANOVA). Student’s t-test was used to identify differences between two groups. A p value <*0.05* was considered statistically significant.

## Results

### Repeated IMQ-induced recurrent psoriatic mice

After treating with repeated IMQ treatment (**Figure 1A**), obvious papulosquamous plaques and bleeding were observed on the backs of mice, along with severe epidermal hyperplasia (**Figure 1B**). Mice in the IMQ–IMQ group also showed higher PASI scores than the control group (**Figure 1C**). Histological analysis further showed that mice in the IMQ–IMQ group mice also displayed severe acanthosis and papillomatosis (**Figure 1C**). These data indicate that recurrent psoriatic mice were successfully established. Meanwhile, cutaneous inflammatory cytokines (IL-17A, IL-6, and TNF-α) were elevated in the IMQ–IMQ group (**Figure 1C**). These findings suggest that psoriasiform dermatitis emerges in repeated IMQ-induced recurrent psoriatic mice.

### T memory cells in spleen and LNs of primary psoriatic mice

**Figure 2:**
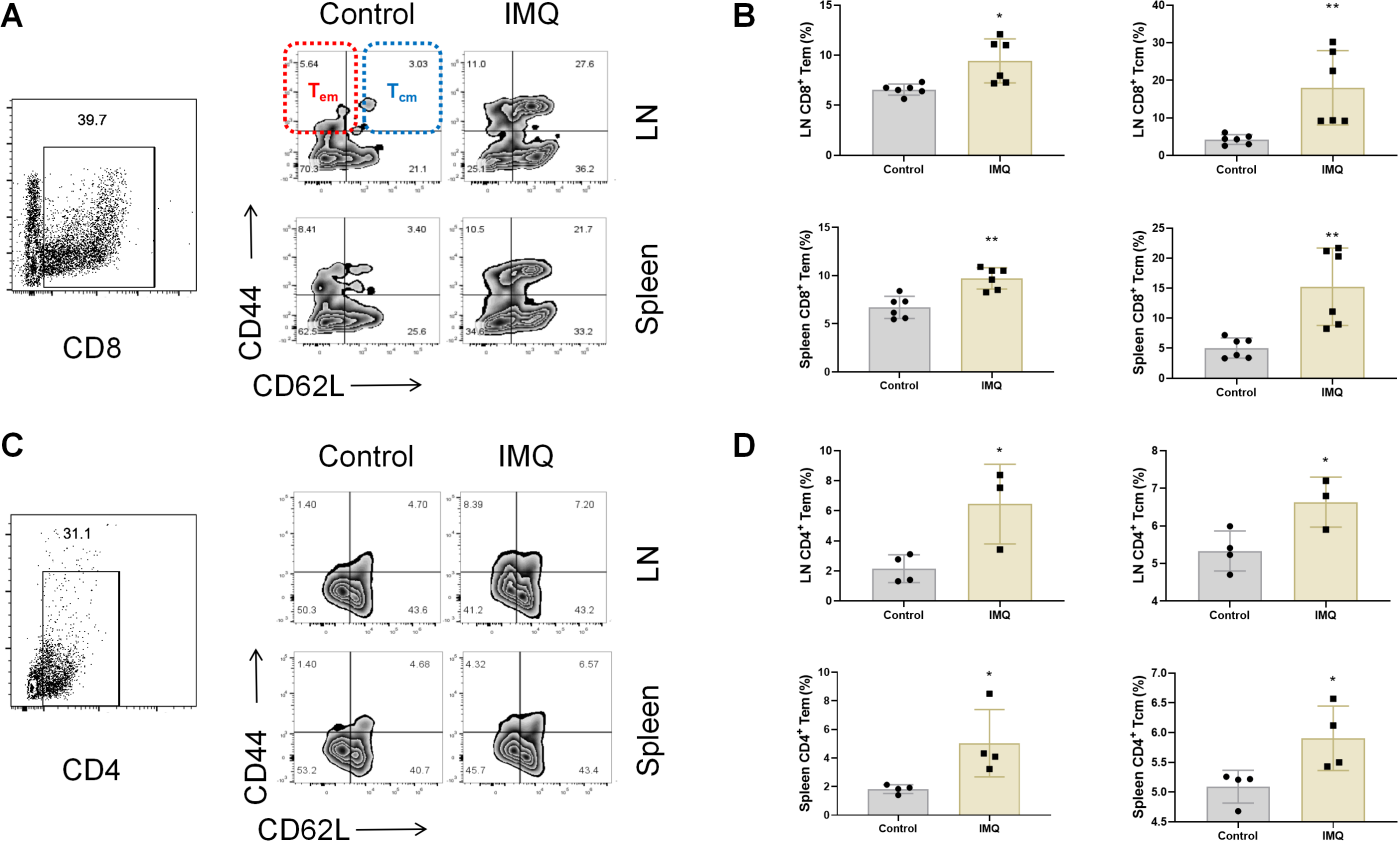
Memory T cells in IMQ-induced primary psoriatic mice. Representative dot plots of CD8^+^ central memory T (Tcm) cells and effector memory T (Tem) cells from lymph nodes (LNs) and spleen of psoriatic mice **(A)**, average percentages of CD8^+^ CD44^+^ CD62L^+^ Tcm cells and CD8^+^ CD44^+^ CD62L^+^ Tem cells **(B)**, representative dot plots of CD4^+^ Tcm cells and Tem cells from LNs and spleen of psoriatic mice **(C)**, and average percentages of CD4^+^ CD44^+^ CD62L^+^ Tcm cells and CD4^+^ CD44^+^ CD62L^-^ Tem cells **(D)**. Values are means ± SD, n = 6 per group for **(A, B)** and n = 3–4 per group for **(C, D)**. *P<0.05, **P<0.01 versus control group.

To investigate the phenotype of T memory cells, Tcm and Tem cells were determined in the Tcm and Tem in spleen and LNs of IMQ-induced primary psoriatic mice on day 7. Both CD8^+^ Tcm and Tem cells were increased in the spleen and LNs of primary psoriatic mice on day 7 (**Figures 2A, B**). Similarly, CD4^+^ Tcm and Tem cells were also increased in the spleen and LNs of psoriatic mice (**Figures 2C, D**). These data indicate that both CD4^+^ and CD8^+^ T memory cells are activated in primary psoriasis.

### Cutaneous Trm cells in recurrent psoriatic mice

**Figure 3:**
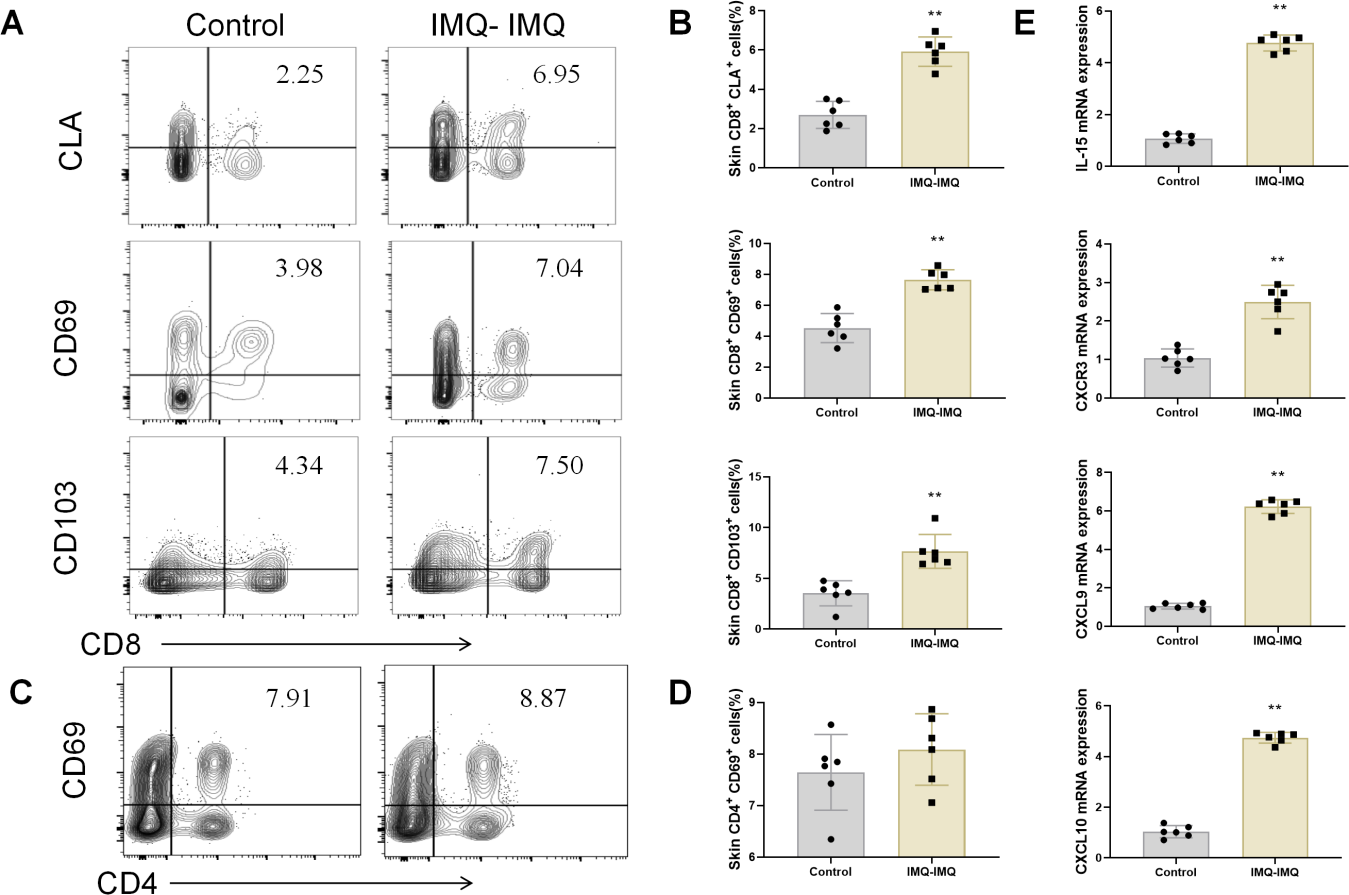
Tissue-resident memory T (Trm) cells in IMQ-induced recurrent psoriatic mice. Representative dot plots of CD8^+^ Trm cells **(A)** and average percentages of CD8^+^ CLA^+^, CD8^+^ CD69^+^, and CD8^+^ CD103^+^ Trm cells **(B)** from the skin of psoriatic mice. Representative dot plots of CD4^+^ Trm cells **(C)** and average percentages of CD4^+^ CD69^+^ Trm cells **(D)** from the skin of psoriatic mice. Cutaneous gene expression of IL-15, CXCR3, CXCL9, and CXCL10 in psoriatic mice **(E)**. Values are means ± SD, n = 6 per group. *P<0.05, **P<0.01 versus control group.

Tem and Tcm cells can further differentiate into Trm cells and persist in the skin (18). After verifying the Tem and Tcm cells in primary psoriatic mice, we further explored the Trm cells in the skin of recurrent psoriatic mice. CD103, CD69, and CLA are biomarkers of Trm cells. The ratio of CD8^+^/CD4^+^ cells was significantly increased in the skin of recurrent psoriatic mice (**Supplementary Figure 1B**). Furthermore, the CD8^+^ CD103^+^, CD8^+^ CD69^+,^and CD8^+^ CLA^+^ Trm cells both were all increased in the skin of IMQ–IMQ-induced recurrent psoriatic mice (**Figures 3A, B**). Interestingly, there was no significant difference in CD4^+^ CD69^+^ Trm cells between the control group and IMQ–IMQ groups (**Figures 3C, D**). These data suggest that CD8^+^ Trm cells, but not CD4^+^ Trm cells, are more important in recurrent psoriasis. This finding is consistent with previous research showing that CD8^+^ Trm cells generate IL-17A to promote local skin inflammation in psoriasis (19). In addition, cutaneous gene expression of IL-15, CXCR3, CXCL9, and CXCL10 was upregulated in recurrent psoriatic mice (**Figure 3E**). These genes are associated with T cell recruitment and Trm cell generation (3, 20–22). Hence, we speculate that CD8^+^ Trm cells are activated and contribute to the recurrence of psoriasiform dermatitis.

### Treating recurrent psoriatic mice with CD8^+^ Tcm, CD4^+^ Tcm cells or rIL-15

**Figure 4:**
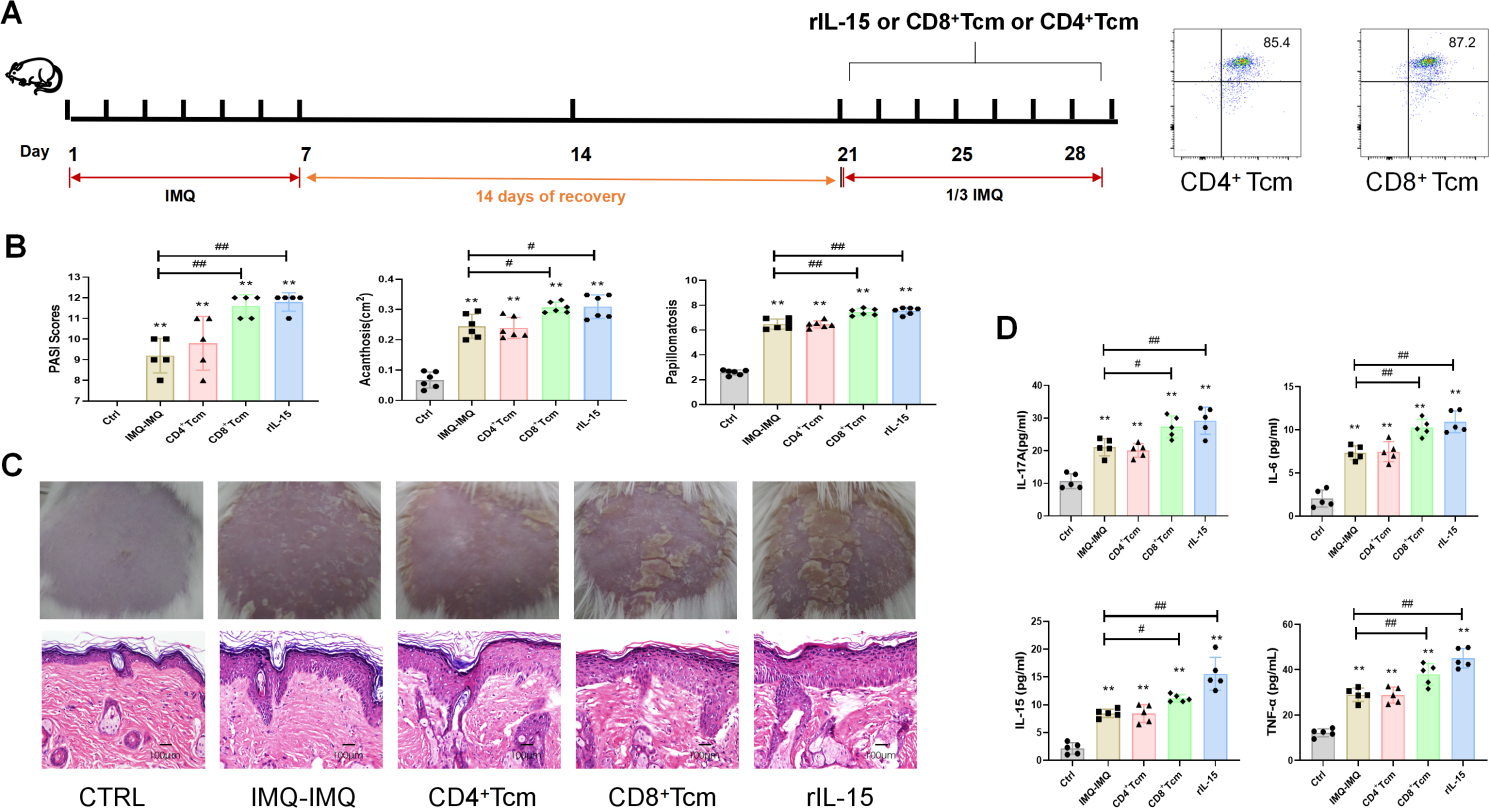
Effects of CD8^+^ Tcm and CD4^+^ Tcm cells in recurrent psoriatic mice. Flow chart of CD8^+^ Tcm/CD4^+^ Tcm or recombinant IL-15 treatment in recurrent psoriatic mice **(A)**, PASI scores, acanthosis, and papillomatosis of recurrent psoriatic mice **(B)**, representative macroscopic images and H&E-stained dorsal skin sections **(C)**, and cutaneous IL-17A, IL-6, IL-15, and TNF-α levels in psoriatic mice **(D)**. Values are means ± SD, n = 5–6 per group. *P<0.05, **P<0.01 versus control group; ^#^*P<0.05*,^##^*P<0.01* versus IMQ–IMQ group.

To validate the effects of CD8^+^ memory cells in recurrent psoriasis, we separated the CD8^+^ Tcm and CD4^+^ Tcm cells were isolated from primary psoriatic mice and injected into recurrent psoriatic mice (**Figure 4A**). We aimed to investigate whether CD8^+^ or CD4^+^ Tcm cells will promote recurrent psoriasis. After injection of CD8^+^ Tcm cells, mice exhibited more severe psoriatic symptoms than IMQ–IMQ-induced recurrent psoriatic mice (**Figures 4B, C**). Cutaneous IL-17A, IL-15, IL-6, and TNF-α levels were also higher in the CD8^+^ Tcm-treated group than in IMQ–IMQ-induced recurrent psoriatic mice (**Figure 4D**). However, injection of CD4^+^ Tcm cells resulted in psoriatic symptoms similar to those observed in IMQ–IMQ-induced recurrent psoriatic mice (**Figures 4B–D**). Meanwhile, mice treated with rIL-15 also exhibited psoriasiform dermatitis, high PASI scores, acanthosis, papillomatosis, and elevated inflammatory cytokines, comparable to the CD8^+^ Tcm group (**Figures 4B–D**). IL-15 is a key cytokine that promotes the generation and survival of Trm cells in the skin (23–27). Therefore, these findings indicate that IL-15–associated CD8^+^ Trm cells, but not CD4^+^ Trm cells, promote the recurrent psoriasis.

### Canonical and non-canonical NF-κB in recurrent psoriatic mice

**Figure 5:**
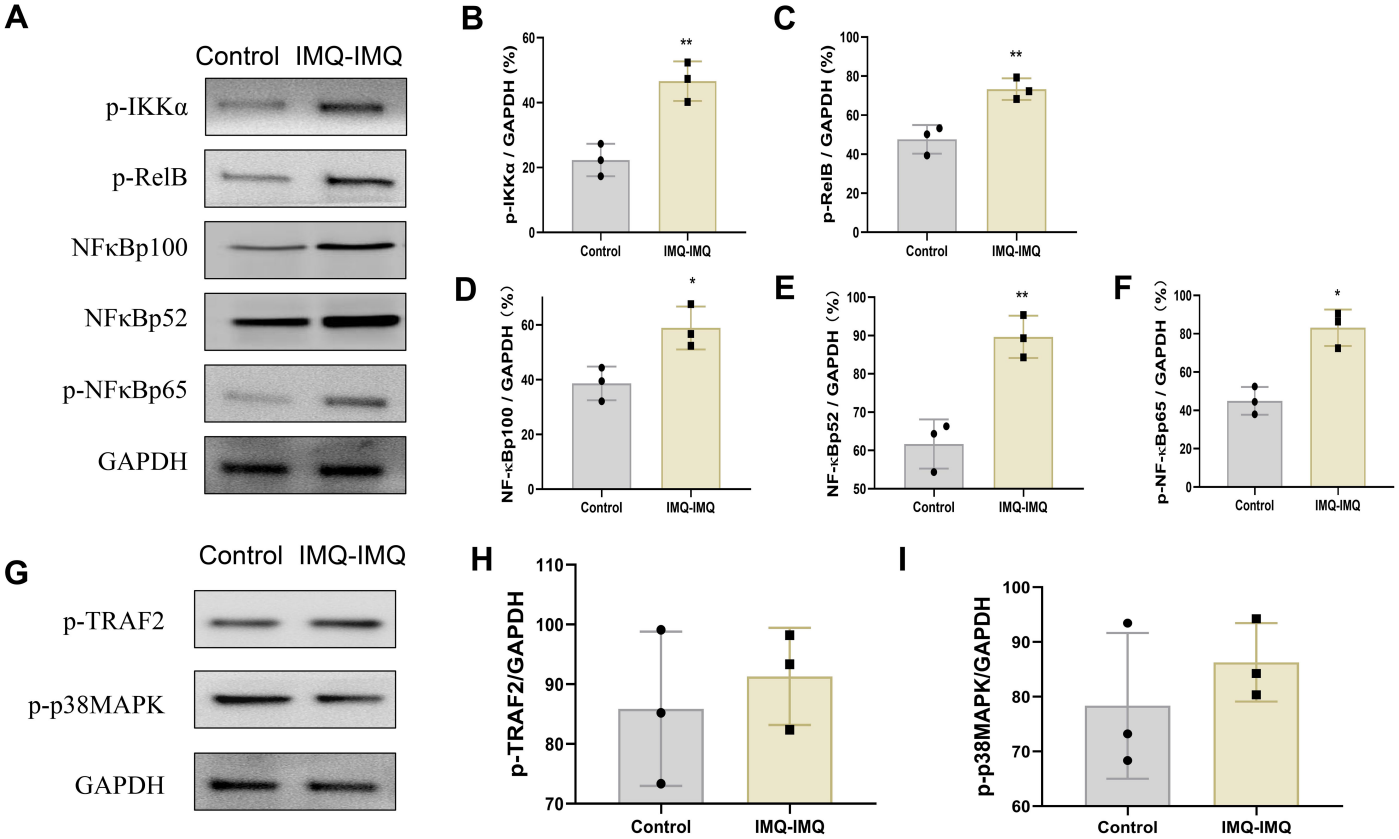
Protein expression of NF-κB signaling in IMQ induced recurrent psoriatic mice. Protein bands of p-IKK, p-RelB, NF-κB p100/p52 and p-NF-κB p65 **(A)**, average phosphorylated level of p-IKKα**(B)**, p-RelB **(C)**, NF-κB p100 **(D)**, NF-κB p52 **(E)**and p-NF-κB p65 **(F)**, protein bands of p-TRAF2 and p-p38MAPK **(G)**, average level of p-TRAF2 **(H)** and p-p38MAPK **(I)**. Values are means ± SD, n = 3 per group. *P<0.05, **P<0.01 versus control group.

To explore the mechanisms underlying Trm cell activation in recurrent psoriasis, the protein expression of classic NF-κB and MAPK were determined. The phosphorylated regulatory factor of canonical NF-κB and MAPK signaling components was examined. Phosphorylated canonical NF-κB p65 (RELA) and non-canonical p-IKKα, p-RelB and NF-κB p100/p52 were up-regulated in IMQ-IMQ induced recurrent psoriatic mice (**Figures 5A–F**). These results suggest that both canonical and non-canonical NF-κB signaling pathways may contribute to activation of Trm cell activation. TRAF2 is a TNF receptor–associated factor that can be activated by TNF family members and functions as an adaptor protein in activating NF-κB activation through various downstream signaling mechanisms, including mitogen-activated protein kinase (MAPK) pathways (28, 29). However, the phosphorylation levels of p38 MAPK and TRAF2 in the skin were similar between the control group and IMQ–IMQ groups (**Figures 5G–I**).

### Treating recurrent psoriatic mice with NF-κB inhibitor and agonist

**Figure 6:**
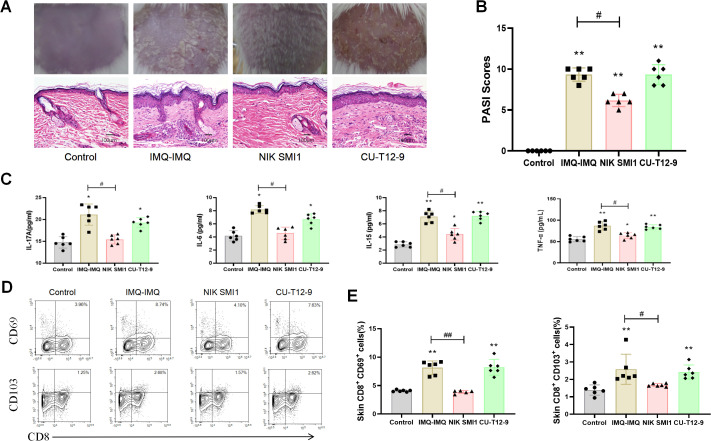
Effects of NF-κB inhibitor and agonist in IMQ-induced recurrent psoriatic mice. Representative macroscopic images and H&E-stained dorsal skin sections **(A)**, PASI scores **(B)**, cutaneous IL-17A, IL-6, IL-15, and TNF-α levels **(C)**, representative dot plots of CD8^+^ Trm cells **(D)**, and average percentages of CD8^+^ CD69^+^ and CD8^+^ CD103^+^ Trm cells **(E)** in the skin of recurrent psoriatic mice. Values are means ± SD, n = 6 per group. *P<0.05, **P<0.01 versus control group. ^#^*P<0.05*, ^##^*P<0.01* versus IMQ–IMQ group.

**Figure 7:**
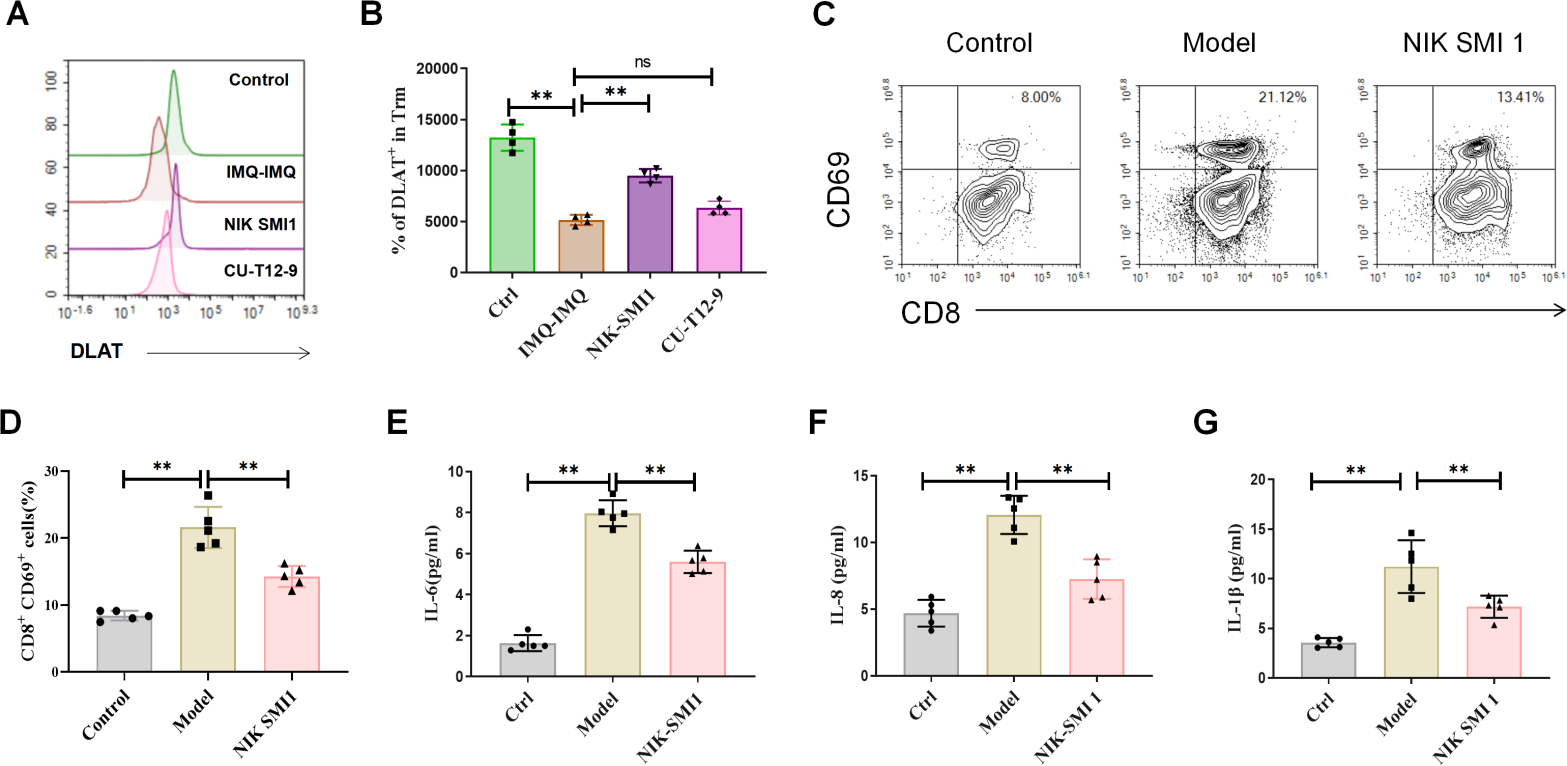
Direct effects of NF-κB on Trm cells. Fluorescence intensity of DLAT fluorescence **(A)** and percentage of DLAT^+^ Trm cells **(B)** in recurrent psoriatic mice. Representative dot plots of CD8^+^ Trm cells **(C)**, average percentages of CD8^+^ Trm cells **(D)**, and levels of IL-6 **(E)**, IL-8 **(F)**, and IL-1β **(G)** in a psoriasis-like model *in vitro*. Values are means ± SD, n = 4–6 per group. *P<0.05, **P<0.01 versus the corresponding control group.

To validate the role of NF-κB in CD8^+^ Trm cells and recurrent psoriasis, IMQ–IMQ-induced recurrent psoriatic mice were received an NF-κB inhibitor (NIK SMI 1) or an NF-kB agonist (CU-T12-9) which served as a mechanistic control for NF-κB activation (**Supplementary Figure 1A**). After treatment with NIK SMI 1, the papulosquamous plaques, PASI scores, and epidermal hyperplasia both were all normalized in recurrent psoriatic mice (**Figures 6A, B****;** **Supplementary Figure 1C, D**). Cutaneous IL-17A, IL-6, IL-15, and TNF-α levels were reduced following NIK SMI 1 treatment (**Figure 6C**). In addition, CD8^+^ CD103^+^ and CD8^+^ CD69^+^ Trm cells in the skin also were decreased by NIK SMI 1 treatment (**Figures 6D, E**). In contrast, mice in the CU-T12–9 group did not exhibit more severe psoriatic symptoms than those in the IMQ–IMQ group (**Figures 6A–E**). These data indicate that the generation of CD8^+^ Trm cells and recurrent psoriasis is dependent on NF-κB signaling in IMQ–IMQ-induced psoriatic mice. Meanwhile, the DLAT expression was reduced in Trm cells of recurrent psoriatic mice (**Figures 7A, B**). However, NF-κB inhibitor treatment significantly restored the expression of DLAT expression (**Figures 7A, B**). These findings suggest that NF-κB activation of NF-κB may promote the retention of CD8^+^ Trm cells by inhibiting the cuproptosis.

### Effects of NF-κB in Trm cells *in vitro*

Although the effects of NF-κB signaling on CD8^+^ Trm cells were validated *in vivo*, these experiments were insufficient to elucidate the specific cellular mechanisms of NF-κB in CD8^+^ Trm cells. Therefore, keratinocytes and CD8^+^ T cells were co-cultured to investigate the direct effects and mechanisms of activating NF-κB activation in Trm cells. The IL-17 was used to induce psoriasis-like model in keratinocytes and the recombinant IL-15 and TGF-β were used to induce the CD8^+^ Trm cells differentiation. After 48 h, CD8^+^ Trm cells were significantly reduced following NIK SMI 1 treatment (**Figures 7C, D**). Expression levels of IL-6, IL-8, and IL-1β both were also decreased by NF-κB inhibition in the supernatant (**Figures 7E–G**). These results indicate that inhibition of NF-κB in CD8^+^ T cells suppresses CD8^+^ Trm cell differentiation of CD8^+^ Trm cells and prevent production of pro-inflammatory factors.

## Discussion

T memory cell–triggered inflammatory responses are the primary pathogenic mechanism and challenge in treating recurrent psoriasis following infection or stimulation by previous pathogens. Once naïve T cells are activated by dendritic cells, a fraction of T cells differentiates into Tcm and Tem cells (4). In inflammatory dermatoses, these Tcm and Tem cells can further differentiate into Trm cells and persist in the skin for a long time (18). These cutaneous Trm cells can rapidly respond to previous pathogens and promote the recurrence of psoriasis.

In this study, both CD4^+^ and CD8^+^ Tem and Tcm cells were increased in the spleen and LNs of primary psoriatic mice, indicating that T memory cells are activated and persist in lymphoid organs. These memory cells can further migrate to the skin and differentiate into Trm cells (3, 18). To verify which type of T memory cells contributes to recurrent psoriasis, we established recurrent psoriatic mice using different dosages of IMQ. After mice recovered from the primary psoriasis, only 20.8 mg/day IMQ (one-third of the initial dosage) was sufficient to induce severe psoriasiform dermatitis again, consistent with previous studies (16, 30). This phenotype indicates that T memory cell–associated immunological memory can rapidly respond to minimal antigen stimulation (IMQ) and produce similar dermatitis. These findings validate that Trm-associated immunological memory triggers psoriasis recurrence of psoriasis.

Trm cells express several biomarkers, including CD69, CD103, and CLA (3). CD103 and CLA can bind with E-selectin and P-selectin, promoting retention of Trm cells in the skin (31, 32). In contrast, CD69 attenuates sphingosine-1-phosphate–induced re-circulation to prevent homing of Trm cells (33). Then the blood vasculature system is still disturbed in convalescence after psoriasis and further promote Trm cell retention in the skin (34, 35). In this study, we found that only CD8^+^ Trm cells, but not CD4^+^ Trm cells, were increased in the skin of recurrent psoriatic mice. This finding is consistent with previous reports showing that CD8^+^ Trm cells produce IL-17A and promote local skin inflammation in psoriasis (19). Meanwhile, cutaneous IL-15, CXCR3, CXCL9, and CXCL10 expression was also were enhanced in recurrent psoriatic mice. IL-15 is a crucial cytokine involved in the recruitment and maintenance of Trm cells through multiple signaling pathways (23–27). The CXCR3 facilitates Trm cell generation (20), while CXCL9 and CXCL10 both can activate Trm cells through different mechanisms (21, 22). Together, these results suggest that CD8^+^ Trm cell–associated inflammatory responses are a primary driver of recurrent psoriasis.

To determine which kind of Trm cell population plays a dominant role in recurrent psoriasis, mice were administered CD8^+^ Tcm cells or CD4^+^ Tcm cells isolated from primary psoriatic mice. Mice injected with CD8^+^ Tcm cells displayed more severe psoriasiform dermatitis with injection of CD8^+^ Tcm cells than IMQ–IMQ mice. These results demonstrate that CD8^+^ Trm cells derived from Tcm cells, but not CD4^+^ Tcm cells, trigger the recurrent psoriasis. Meanwhile, rIL-15 treatment induced psoriasiform dermatitis similar to that observed in mice receiving CD8^+^ Tcm cells. In psoriasis, IL-15 is produced by skin keratinocytes, dendritic cells, and fibroblasts and promotes Trm cell activation of Trm cells (36, 37). This finding further validates that IL-15–associated generation of CD8^+^ T memory cells contributes to recurrent psoriasis recurrence.

Elucidating the molecular mechanisms underlying Trm cell activation of Trm is essential for preventing recurrent psoriasis. The NF-κB protein complex is a classic regulator of lymphocyte immune responses. NF-κB activity is regulated by the IκB kinase (IKK) complex. Upon IKK activation, IκB family proteins undergo phosphorylation, ubiquitination, and proteasomal degradation, leading to the release and nuclear translocation of NF-κB family members will be released and nuclear translocated (11). Depending on canonical or non-canonical NF-κB signaling, different subunits are activated. In canonical NF-κB signaling, p105 is processed into p50, p65 (RELA), and c-REL, whereas in non-canonical NF-κB signaling, p100 is processed into p52 and RELB (11). These subunits regulate the generation and maintenance of effector and memory T cells through multiple mechanisms. In vitiligo, reactive oxygen species activate canonical NF-κB signaling in keratinocytes, which secrete IL-15 to activate CD8^+^ Tem cells (38). Based on these findings, we hypothesized that NF-κB signaling may also regulate CD8^+^ Trm cell generation of CD8^+^ Trm cells in recurrent psoriasis. In IMQ–IMQ-induced recurrent psoriatic mice, p-NF-κB p65 (RELA), p-IKKα, p-RelB and NF-κBp100/p52 were upregulated in the skin. However, phosphorylated TRAF2 and pMAPK p38 MAPK levels were unchanged. These results suggest that both canonical and non-canonical NF-κB signaling may contribute to CD8^+^ Trm cell activation of CD8^+^ Trm cells in recurrent psoriasis, although the upstream regulators remain unclear in recurrent psoriasis.

The NF-κB inhibitor (NIK SMI 1) and NF-κB agonist (CU-T12-9) treatments were used to further verify the role of NF-κB signaling in CD8^+^ Trm cells and recurrent psoriasis. The NIK SMI 1 significantly ameliorated psoriatic symptoms in recurrent psoriatic mice and reduced the level of inflammatory cytokine levels and CD8^+^ Trm cell numbers toward control levels, indicating that CD8^+^ Trm cell activity is largely dependent on the NF-κB signaling. In contrast, CU-T12–9 did not exacerbate disease severity or CD8^+^ Trm cell retention, suggesting that NF-κB signaling is already maximally activated in recurrent psoriatic mice. These findings confirm that NF-κB signaling plays a dominant role in CD8^+^ Trm cell–associated recurrent psoriasis recurrence. Meanwhile, expression of the marker of the cuproptosis-related protein DLAT (39) was reduced in CD8^+^ Trm cells and restored by NF-κB inhibition, suggesting that NF-κB activation in CD8^+^ Trm cells may be associated with cuproptosis and mitochondrial energy metabolism.

To further elucidate the cellular mechanisms of NF-κB signaling in Trm cells, the CD8^+^ T cells and keratinocytes were isolated and used to establish an *in vitro* psoriasis-like model. In this *in vitro* model, NF-κB inhibition of NF-κB suppressed the CD8^+^ Trm cell differentiation and attenuated inflammatory responses. These findings indicate that NF-κB signaling in CD8^+^ T cells may induce differentiation of CD8^+^ Trm cells directly promotes CD8^+^ Trm cell differentiation and drives recurrent psoriasis.

In conclusion, activation of canonical and non-canonical NF-κB signaling promotes CD8^+^ Trm cell generation and inhibits cellular cuproptosis of CD8^+^ Trm cells in the skin, thereby inducing recurrent psoriasiform dermatitis in imiquimod-induced psoriatic mice. Targeting this pathway may represent a novel therapeutic strategy for preventing psoriasis recurrence of psoriasis.

## Data Availability

The datasets presented in this study can be found in online repositories. The names of the repository/repositories and accession number(s) can be found in the article/**supplementary material**.

## Supplementary material

Supplementary Figure 1 **(A)** Flow chart of NF-κB inhibitor and agonist treatment in recurrent psoriatic mice. **(B)** Ratio of cutaneous CD8^+^/CD4^+^ cells. **(C)** Acanthosis after treatment with NIK SMI 1 or CU-T12–9 in recurrent psoriatic mice. **(D)** Papillomatosis after treatment with NIK SMI 1 or CU-T12–9 in recurrent psoriatic mice. Values are means ± SD, n = 6 per group. *P<0.05*, *P<0.01* versus control group; ^#^*P<0.05*, ^##^*P<0.01* versus IMQ–IMQ group.
