# Supplementary figures and images for: Activation of NF-κB signaling in tissue-resident memory T cells promotes recurrent psoriasis in mice
Source: Front Immunol. 2026 Feb 9;16:1762269. doi: 10.3389/fimmu.2025.1762269 (PMC12926151; doi:10.3389/fimmu.2025.1762269)

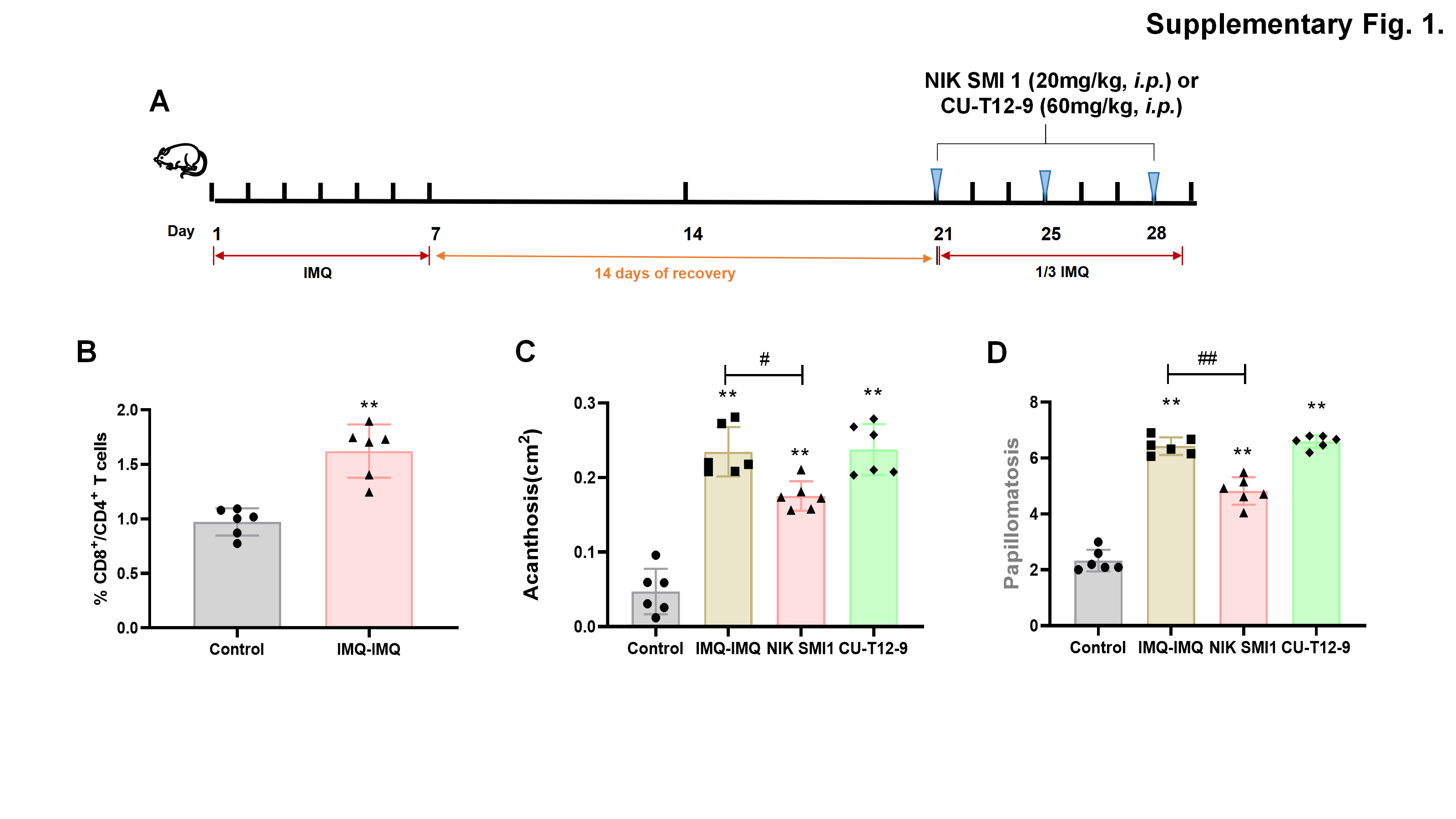

Supplement: Supplementary Figure 1 — (A) Flow chart of NF-κB inhibitor and agonist treatment in recurrent psoriatic mice. (B) Ratio of cutaneous CD8+/CD4+ cells. (C) Acanthosis after treatment with NIK SMI 1 or CU-T12–9 in recurrent psoriatic mice. (D) Papillomatosis after treatment with NIK SMI 1 or CU-T12–9 in recurrent psoriatic mice. Values are means ± SD, n = 6 per group. P<0.05, P<0.01 versus control group; #P<0.05, ##P<0.01 versus IMQ–IMQ group. [file Image1.tif]
