# Supplementary material for: Activation of NF-κB signaling in tissue-resident memory T cells promotes recurrent psoriasis in mice
Source: Front Immunol. 2026 Feb 9;16:1762269. doi: 10.3389/fimmu.2025.1762269 (PMC12926151; doi:10.3389/fimmu.2025.1762269)

Fig 1B

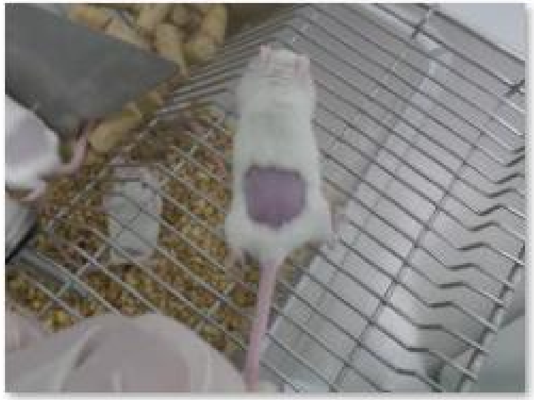
Con1-1

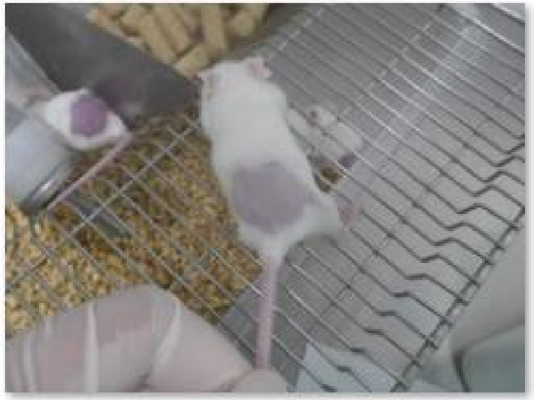
Con2-1

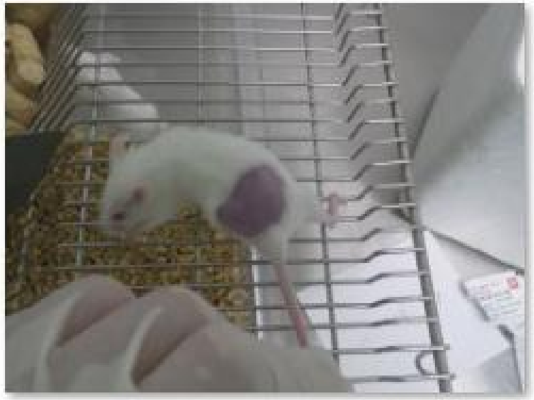
Con3-1

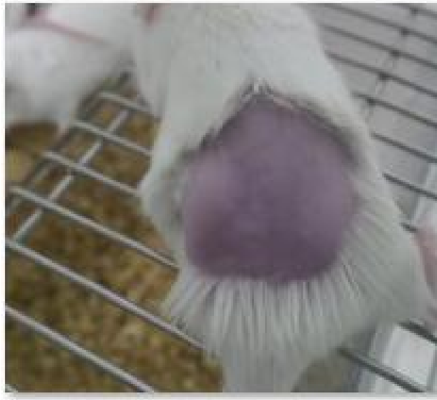
Con4-1

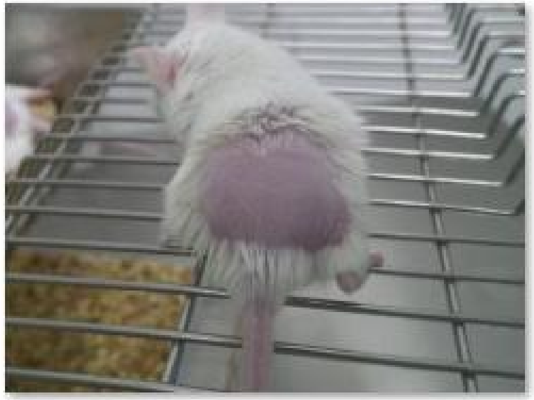
Con5-1

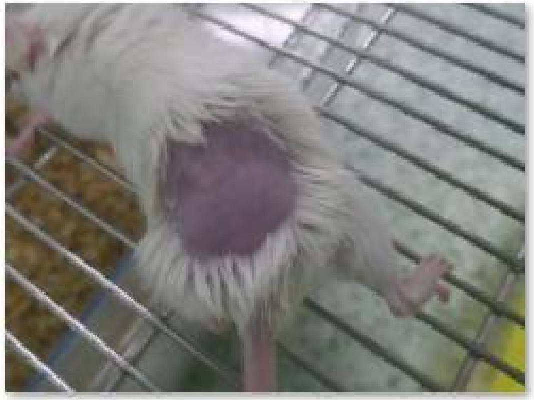
Con6-1

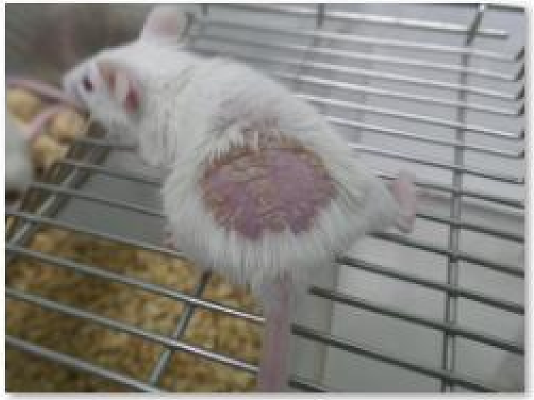
imqimq1-1

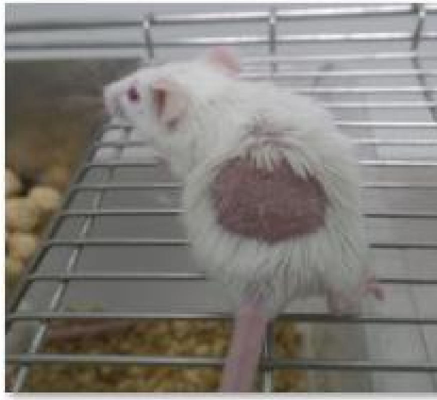
imqimq2-1

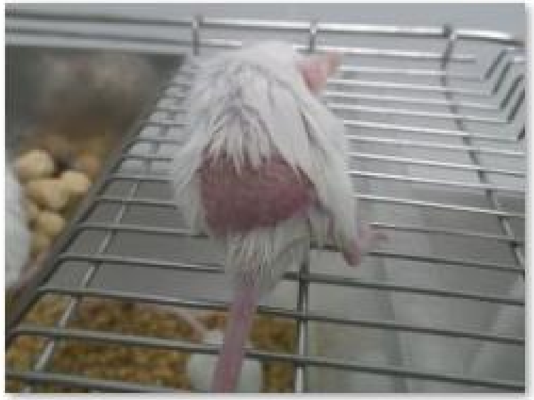
imqimq3-1

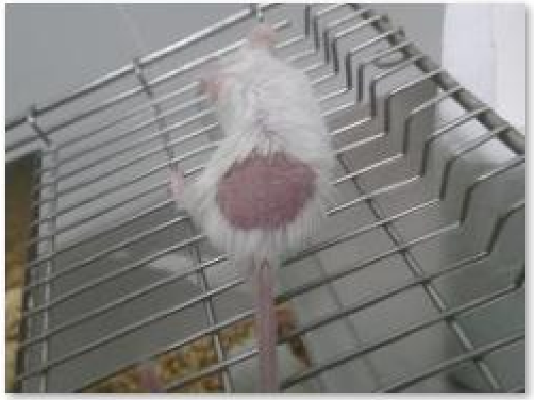
imqimq4-1

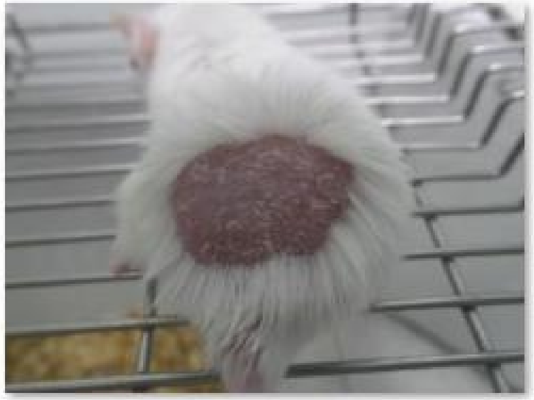
imqimq5-1

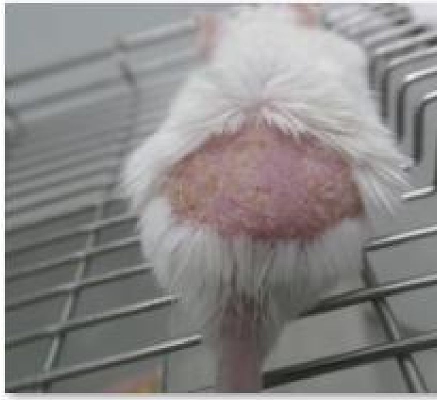
imqimq6-1

Fig 1B

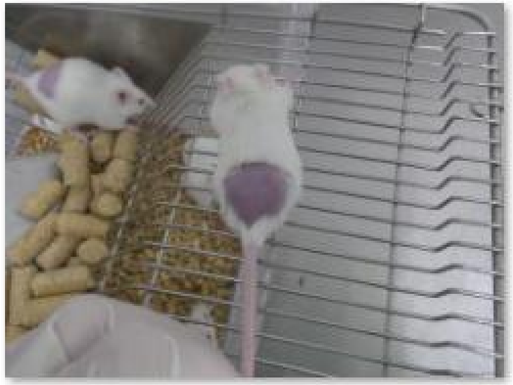

imq-vaselin1-1

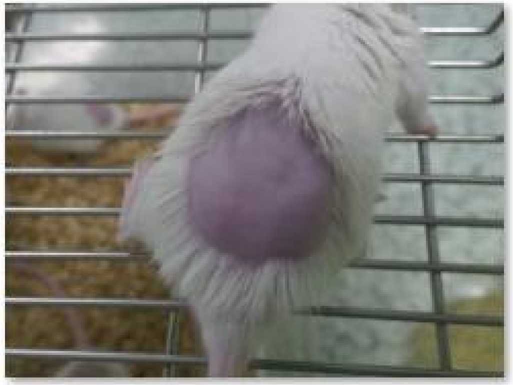

imq-vaselin2-1

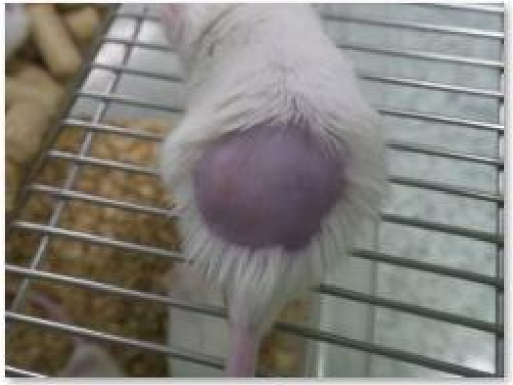

imq-vaselin3-1

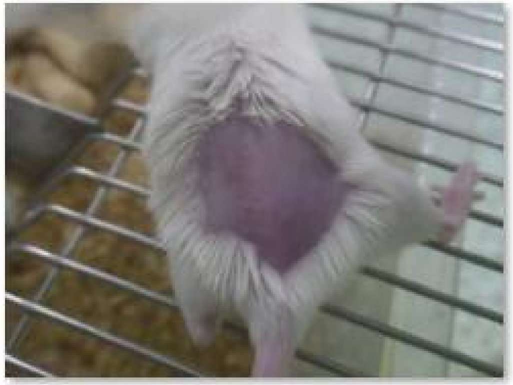

imq-vaselin4-1

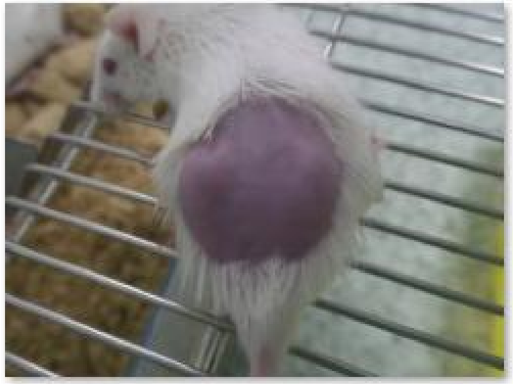

imq-vaselin5-1

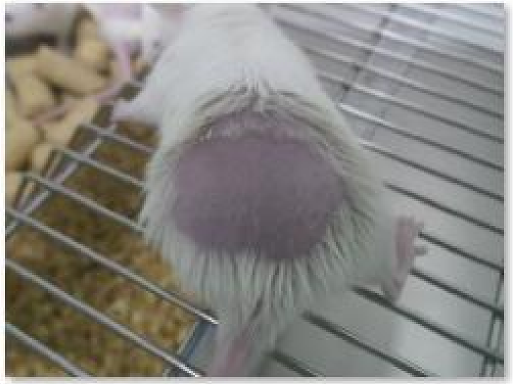

imq-vaselin6-1

Fig 4D

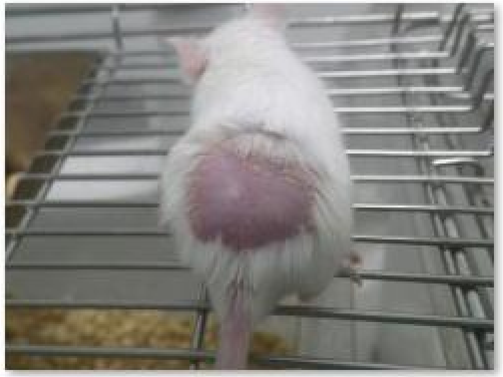

CD4TCM1-2

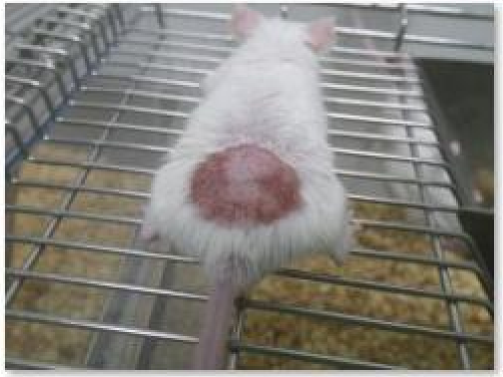

CD4TCM2-2

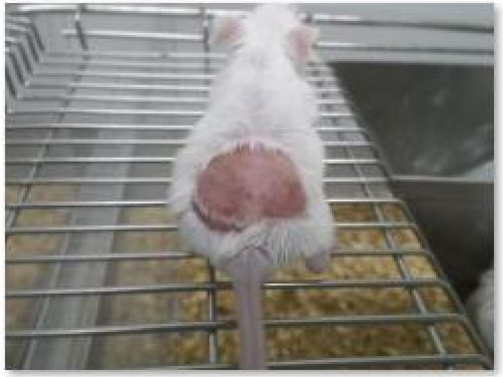

CD4TCM3-2

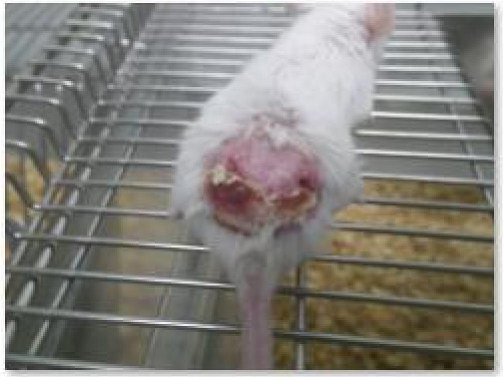

CD4TCM4-2

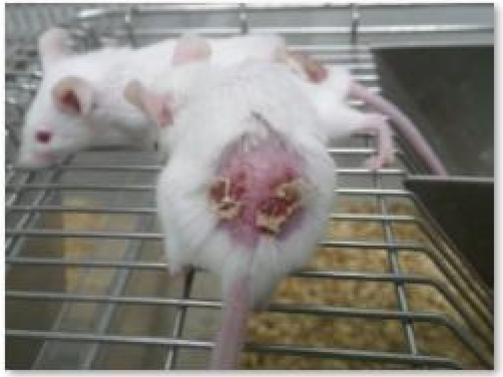

CD4TCM5-2

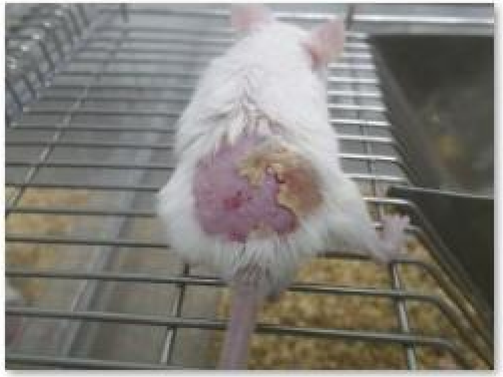

CD4TCM6-2

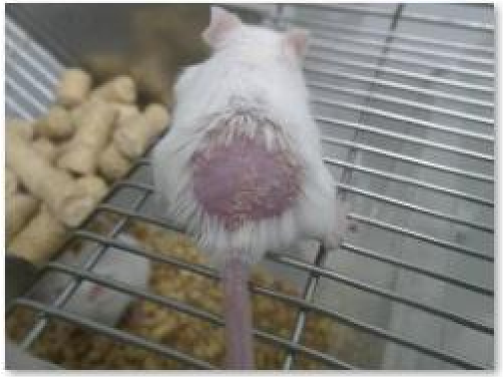

CD8TCM1-2

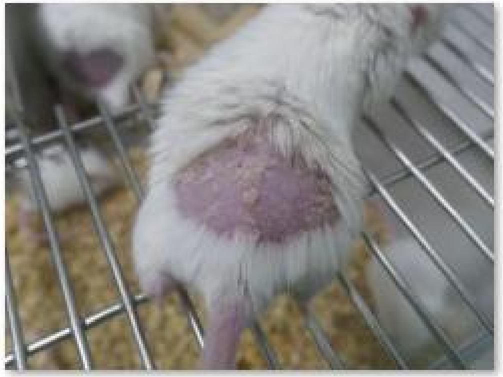

CD8TCM2-2

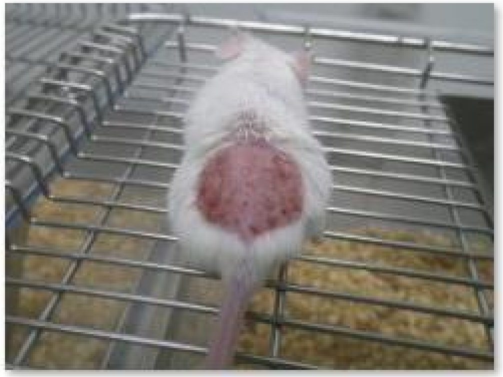

CD8TCM3-2

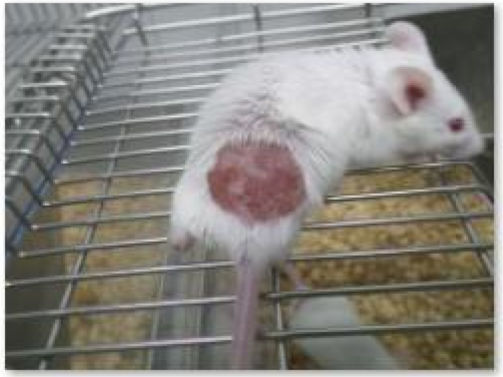

CD8TCM4-2

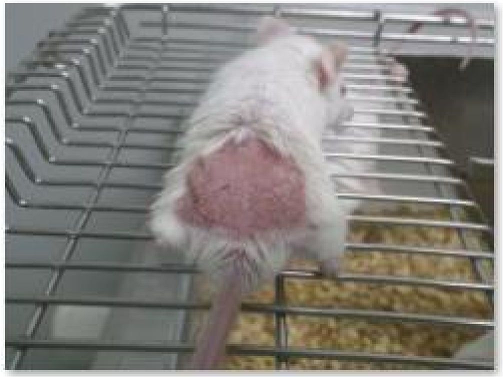

CD8TCM5-2

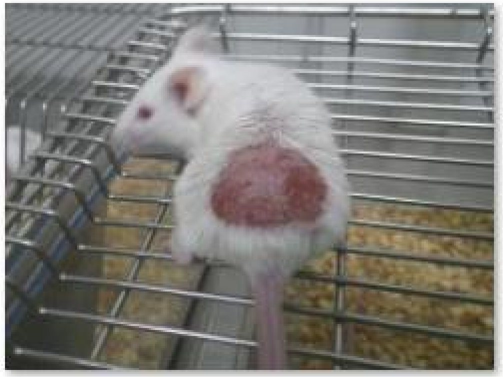

CD8TCM6-2

Fig 4D

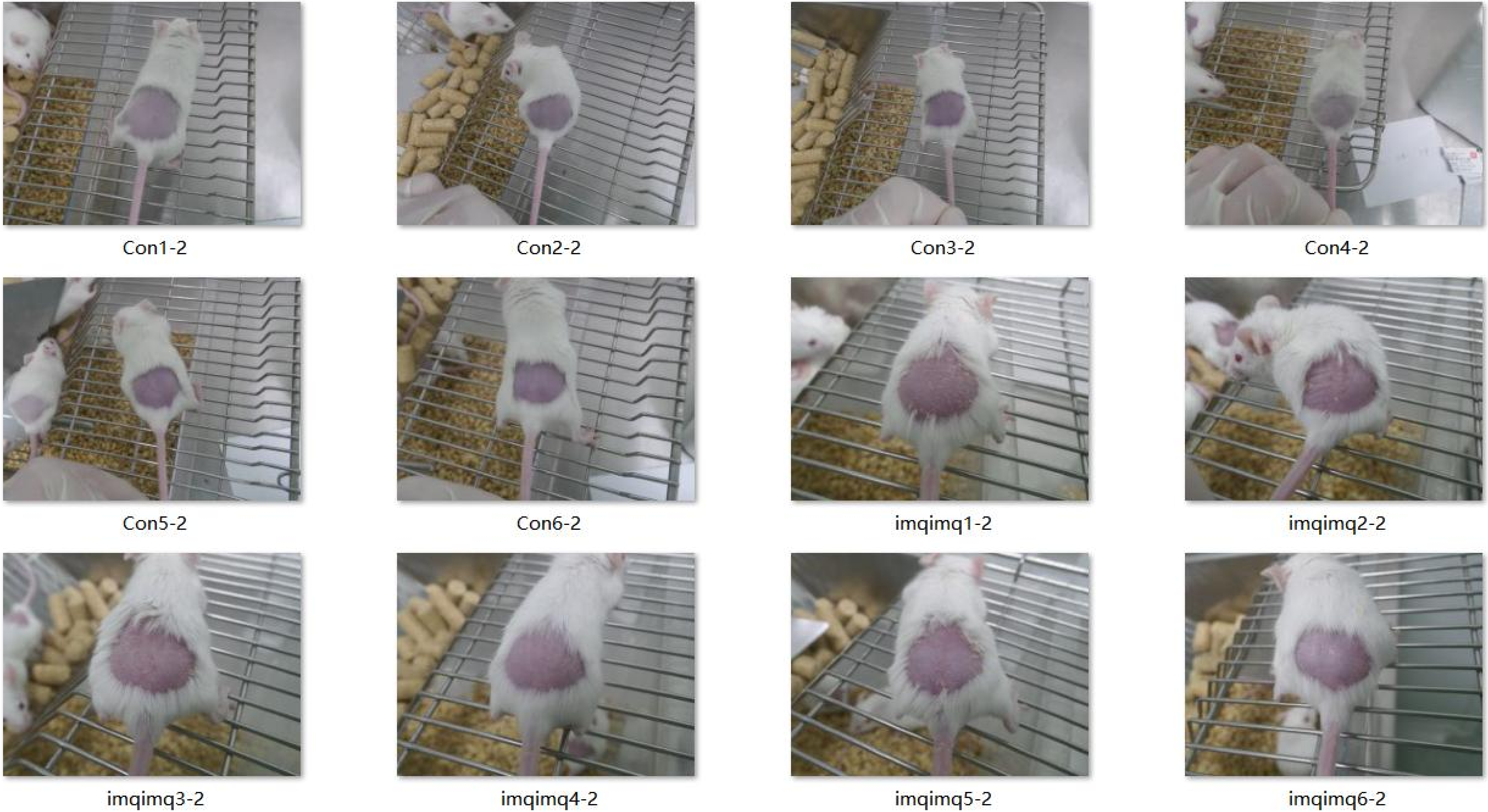
Con1-2
Con2-2
Con3-2
Con4-2
Con5-2
Con6-2
imqimq1-2
imqimq2-2
imqimq3-2
imqimq4-2
imqimq5-2
imqimq6-2

Fig 4D

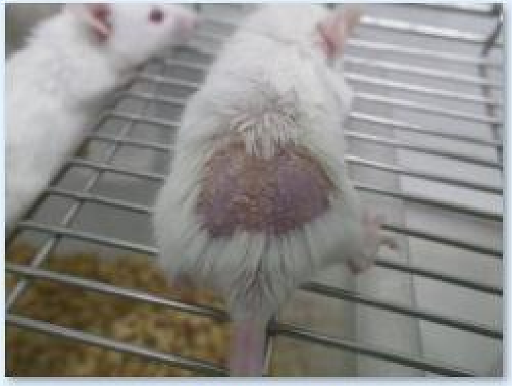

ril15-1-2

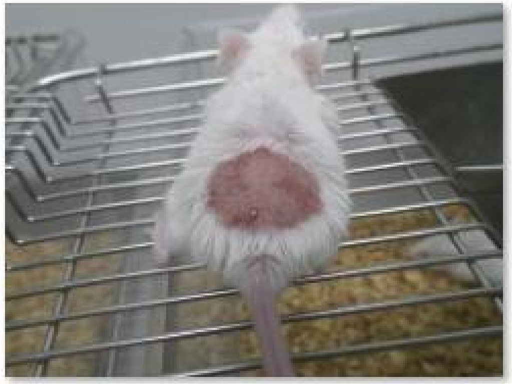

ril15-2-2

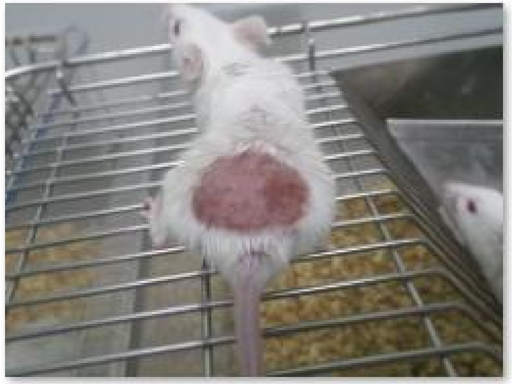

ril15-3-2

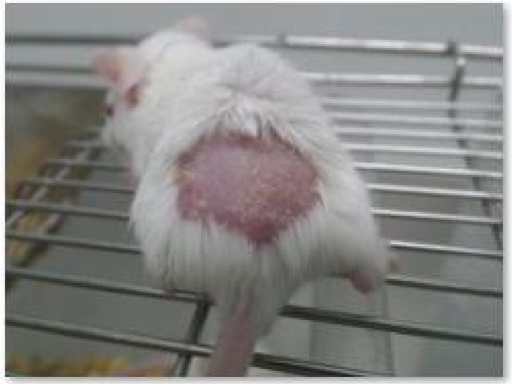

ril15-4-2

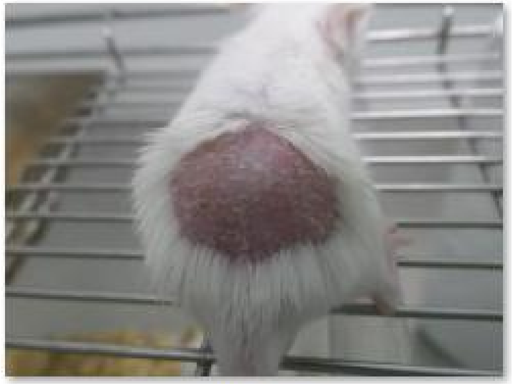

ril15-5-2

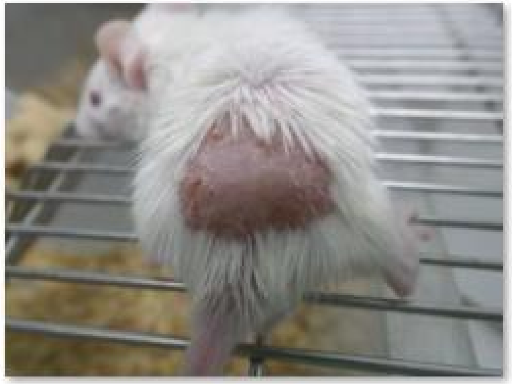

ril15-6-2

Fig 6A

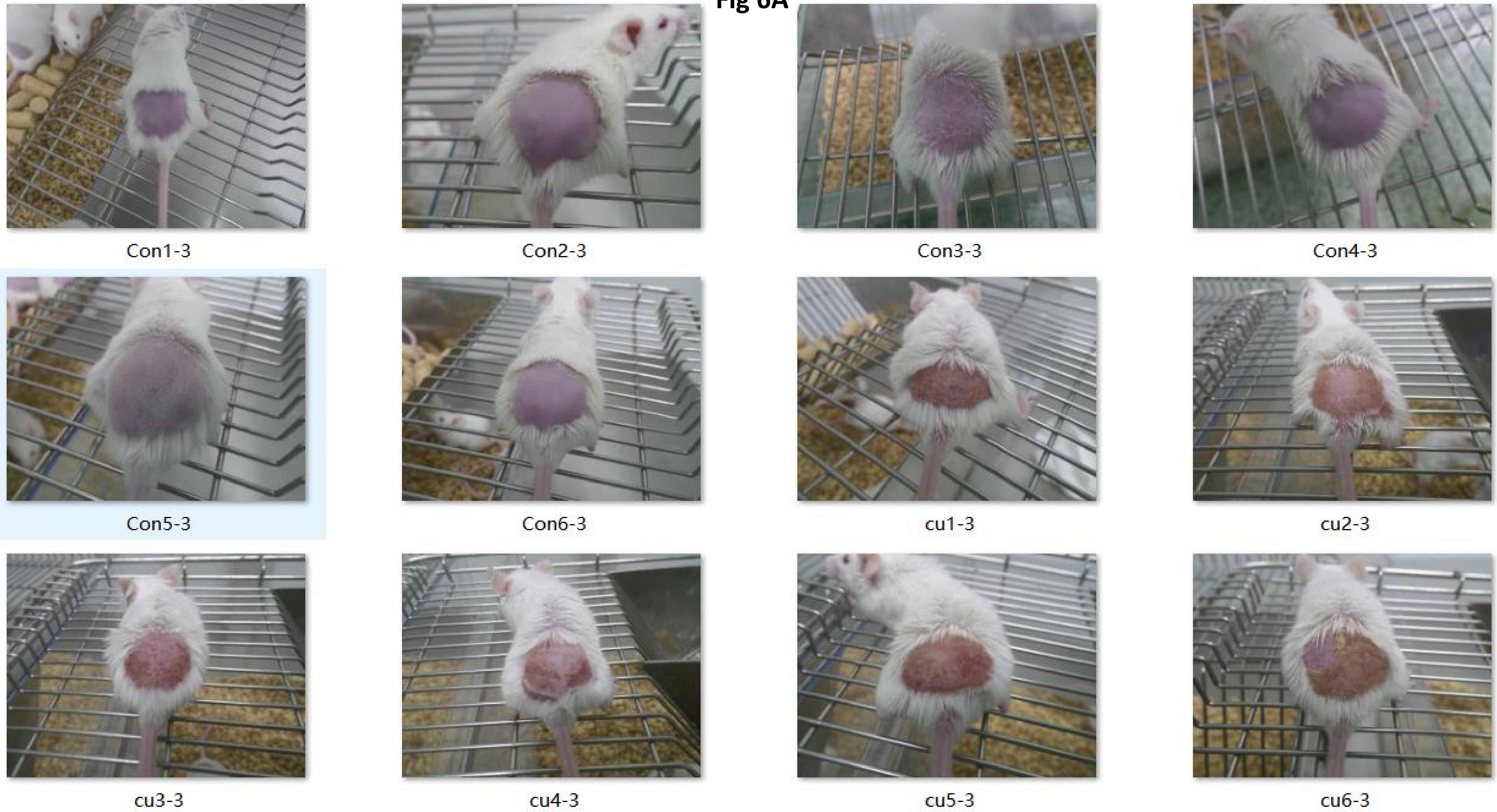
Con1-3
Con2-3
Con3-3
Con4-3
Con5-3
Con6-3
cu1-3
cu2-3
cu3-3
cu4-3
cu5-3
cu6-3

Fig 6A

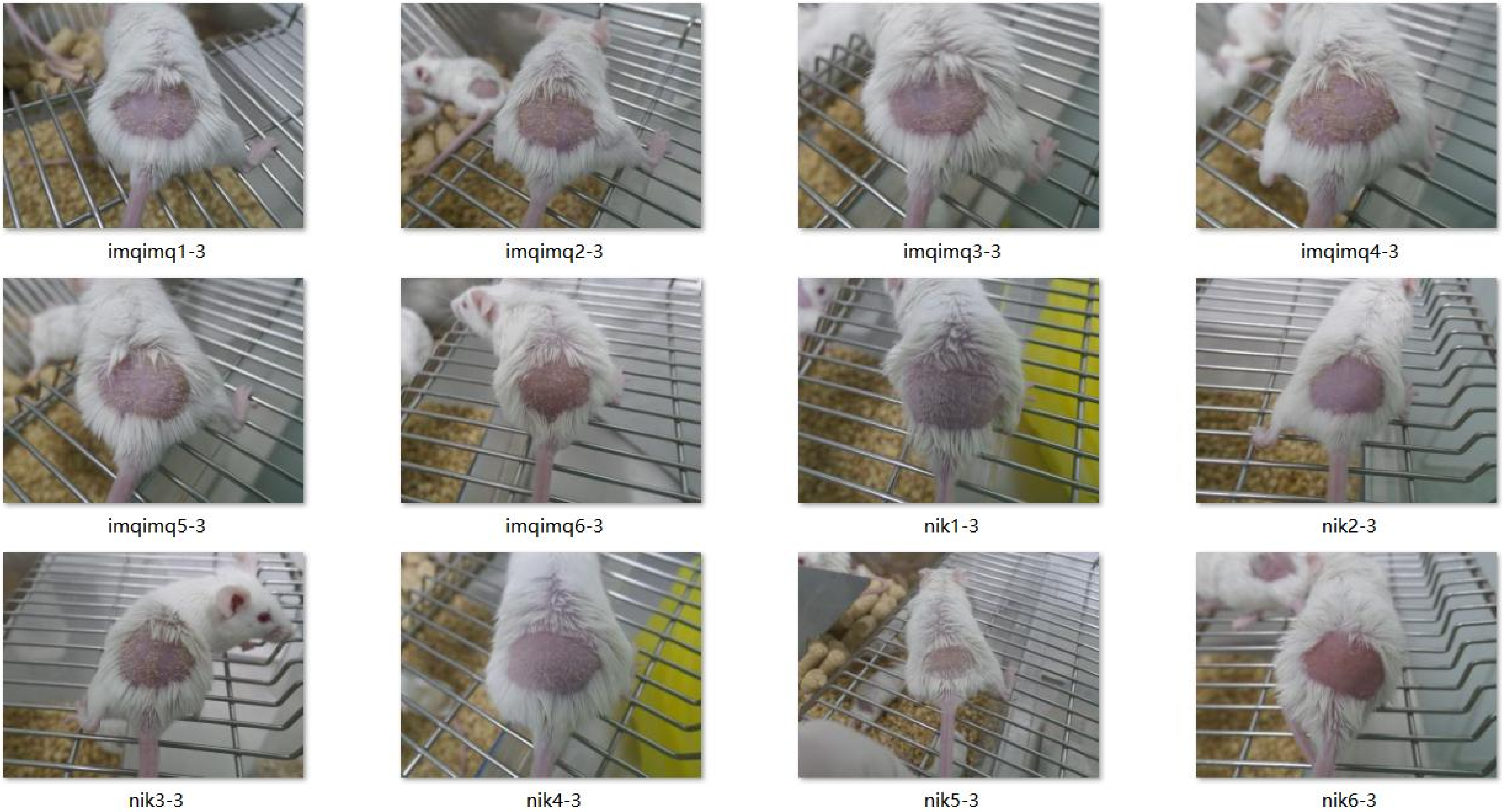
imqimq1-3
imqimq2-3
imqimq3-3
imqimq4-3
imqimq5-3
imqimq6-3
nik1-3
nik2-3
nik3-3
nik4-3
nik5-3
nik6-3

Fig 1B

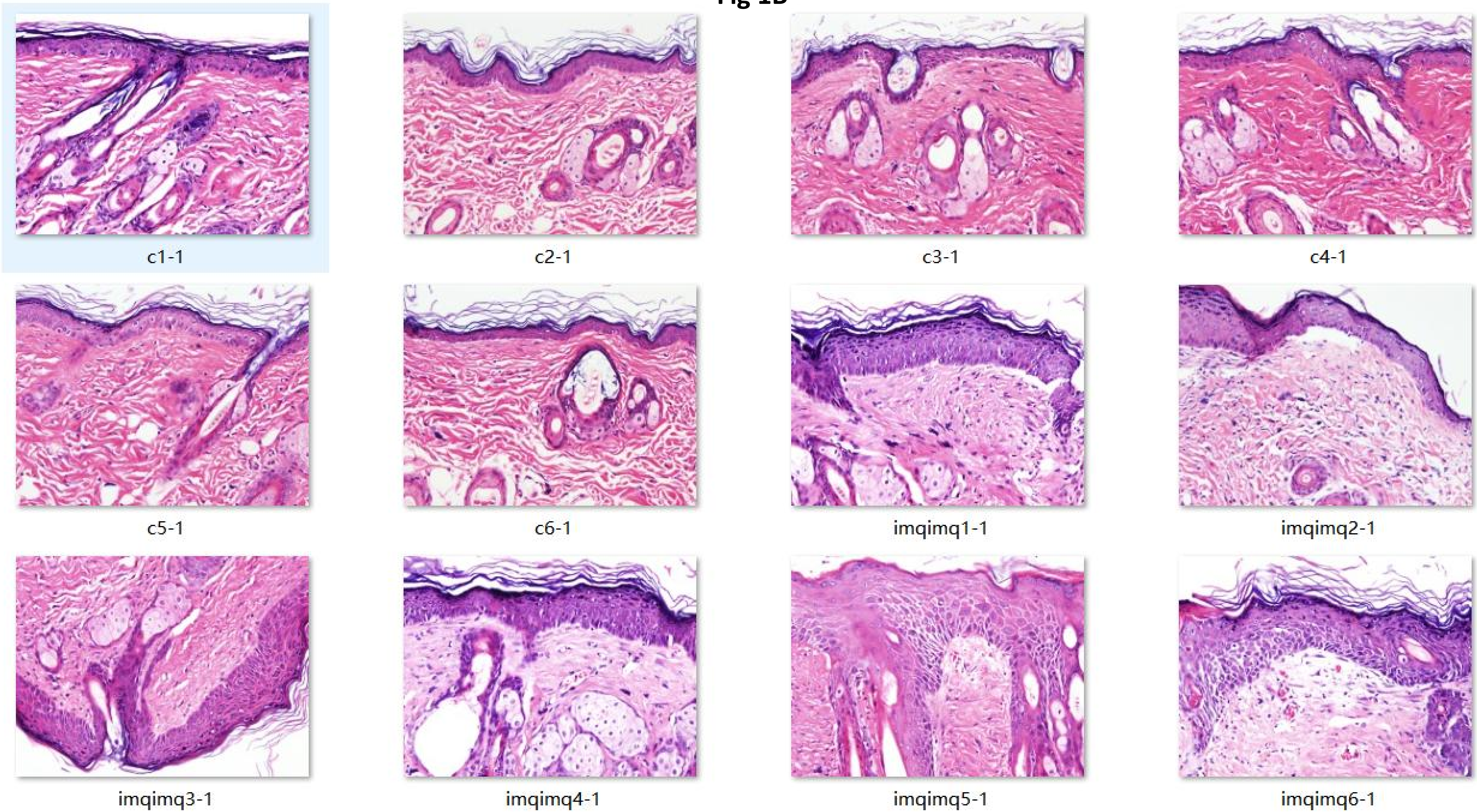
c1-1
c2-1
c3-1
c4-1
c5-1
c6-1
imqimq1-1
imqimq2-1
imqimq3-1
imqimq4-1
imqimq5-1
imqimq6-1

Fig 1B

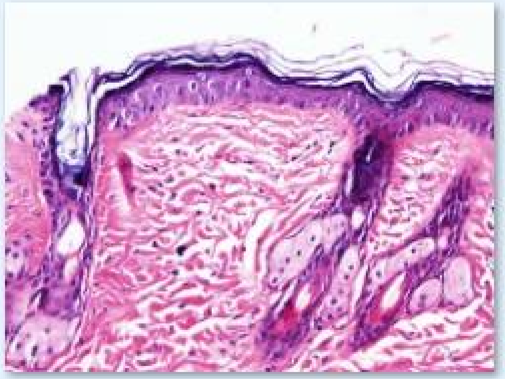

imqvaselin1-1

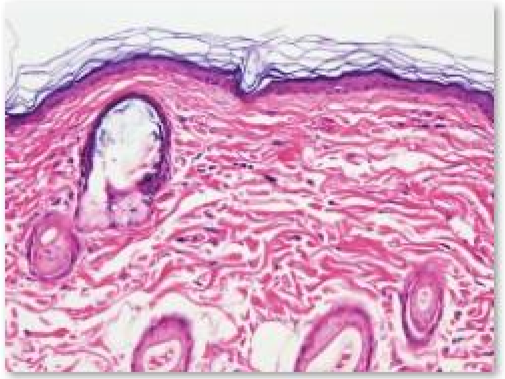

imqvaselin2-1

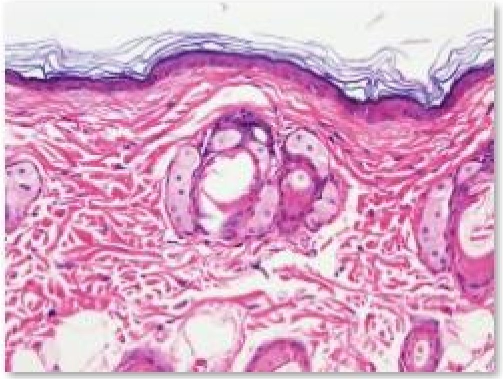

imqvaselin3-1

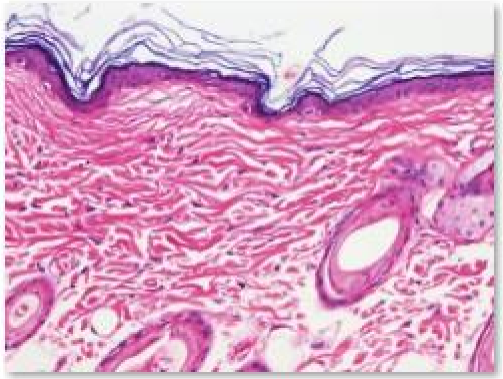

imqvaselin4-1

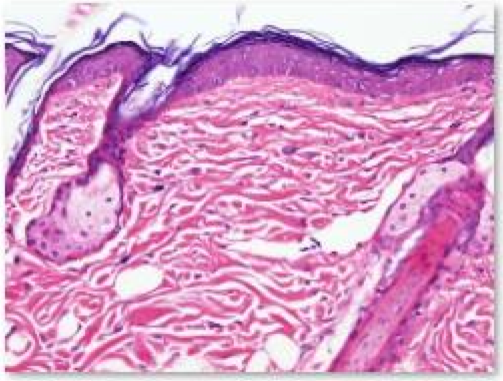

imqvaselin5-1

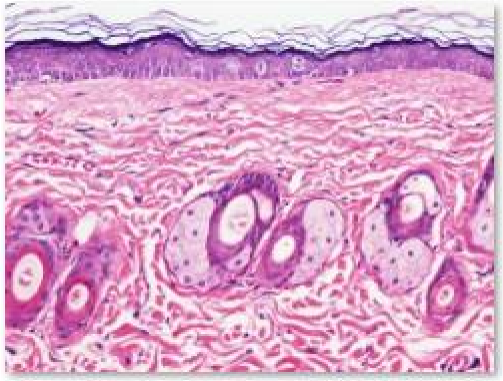

imqvaselin6-1

Fig 4D

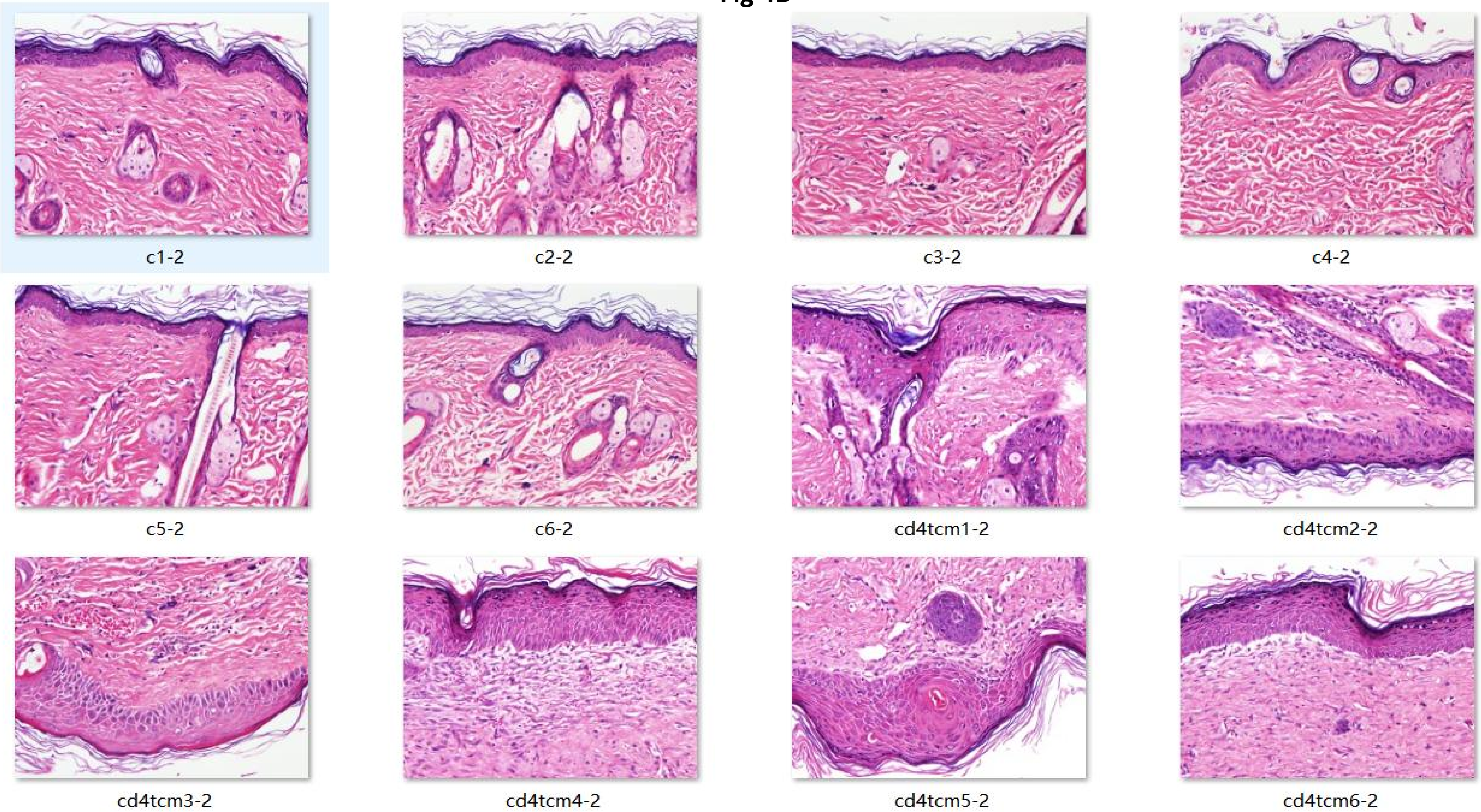
c1-2
c2-2
c3-2
c4-2
c5-2
c6-2
cd4tcm1-2
cd4tcm2-2
cd4tcm3-2
cd4tcm4-2
cd4tcm5-2
cd4tcm6-2

Fig 4D

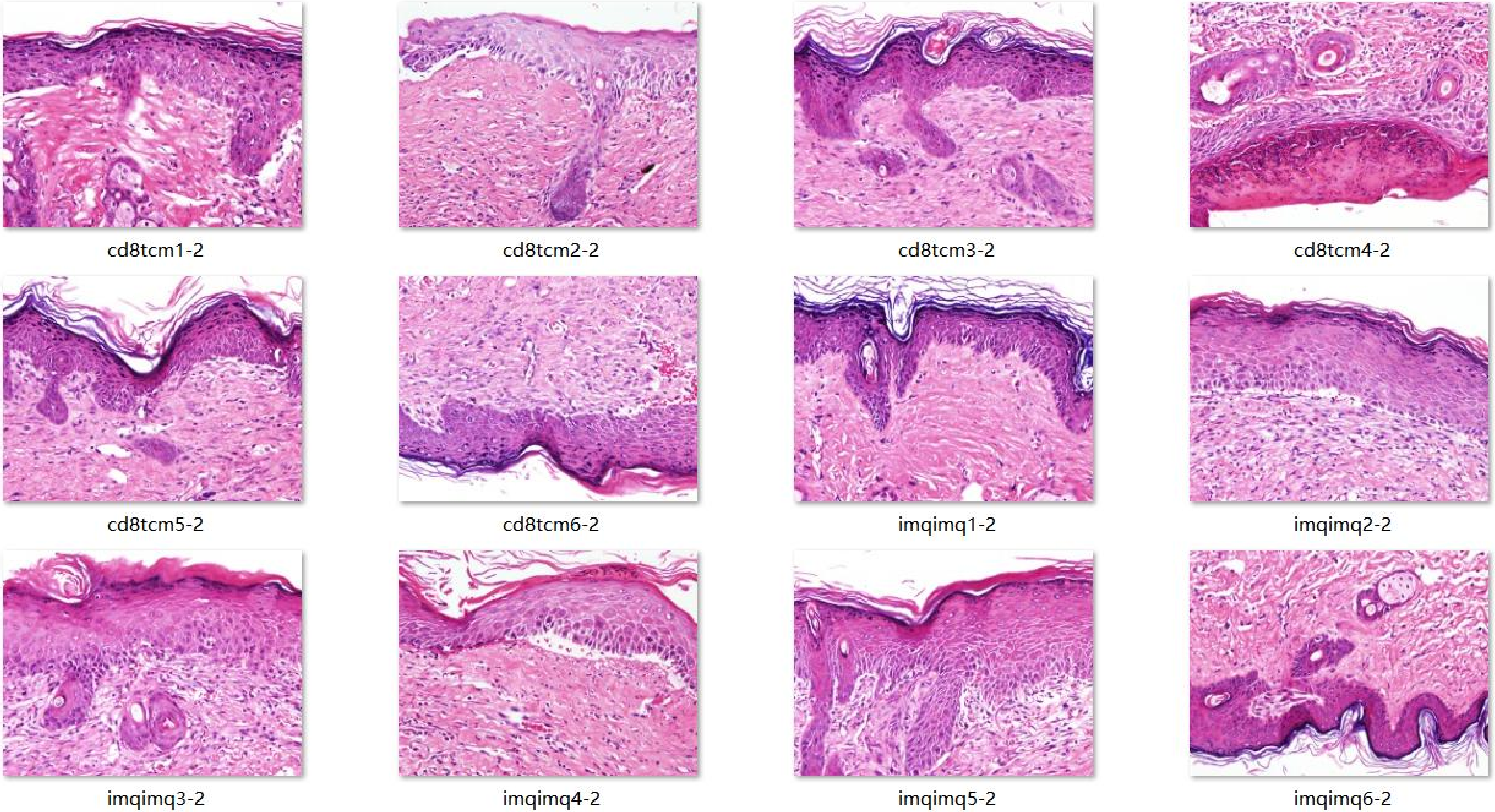
cd8tcm1-2
cd8tcm2-2
cd8tcm3-2
cd8tcm4-2
cd8tcm5-2
cd8tcm6-2
imqimq1-2
imqimq2-2
imqimq3-2
imqimq4-2
imqimq5-2
imqimq6-2

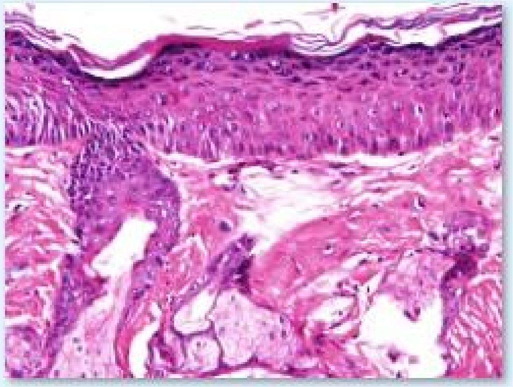
ril15-1-2

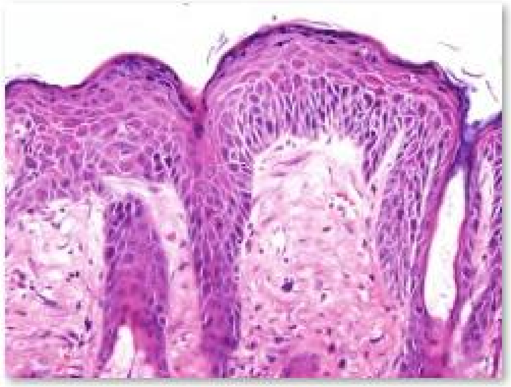
ril15-2-2

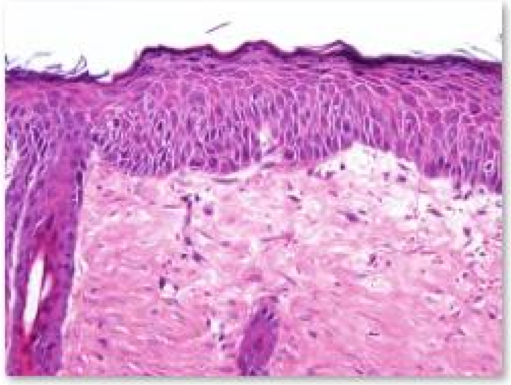
ril15-3-2

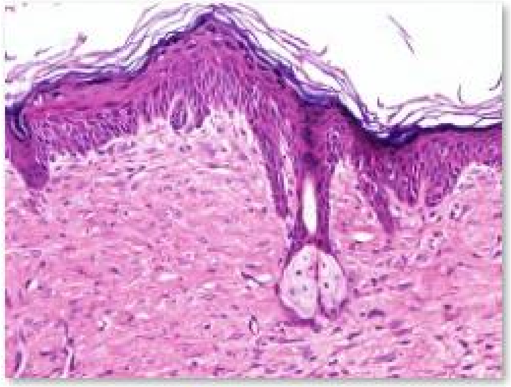
ril15-4-2

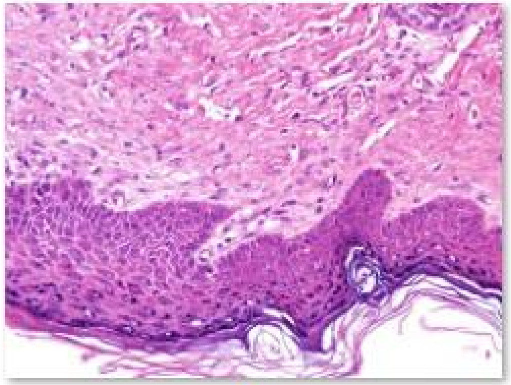
ril15-5-2

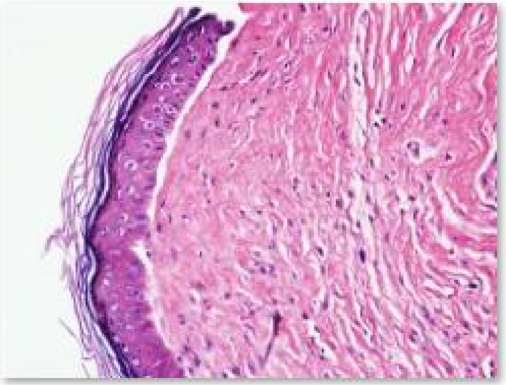
ril15-6-2

Fig 6A

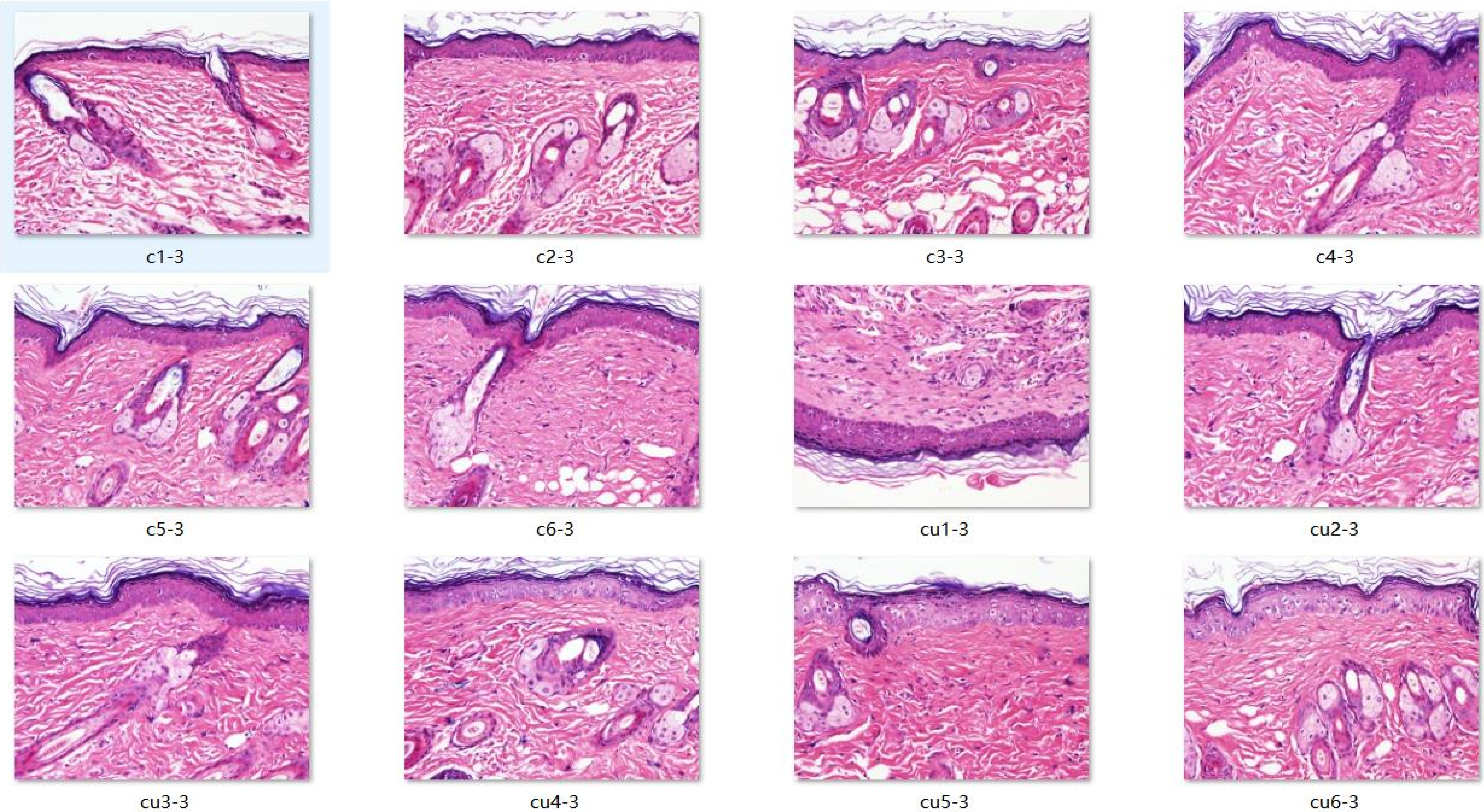

Fig 6A

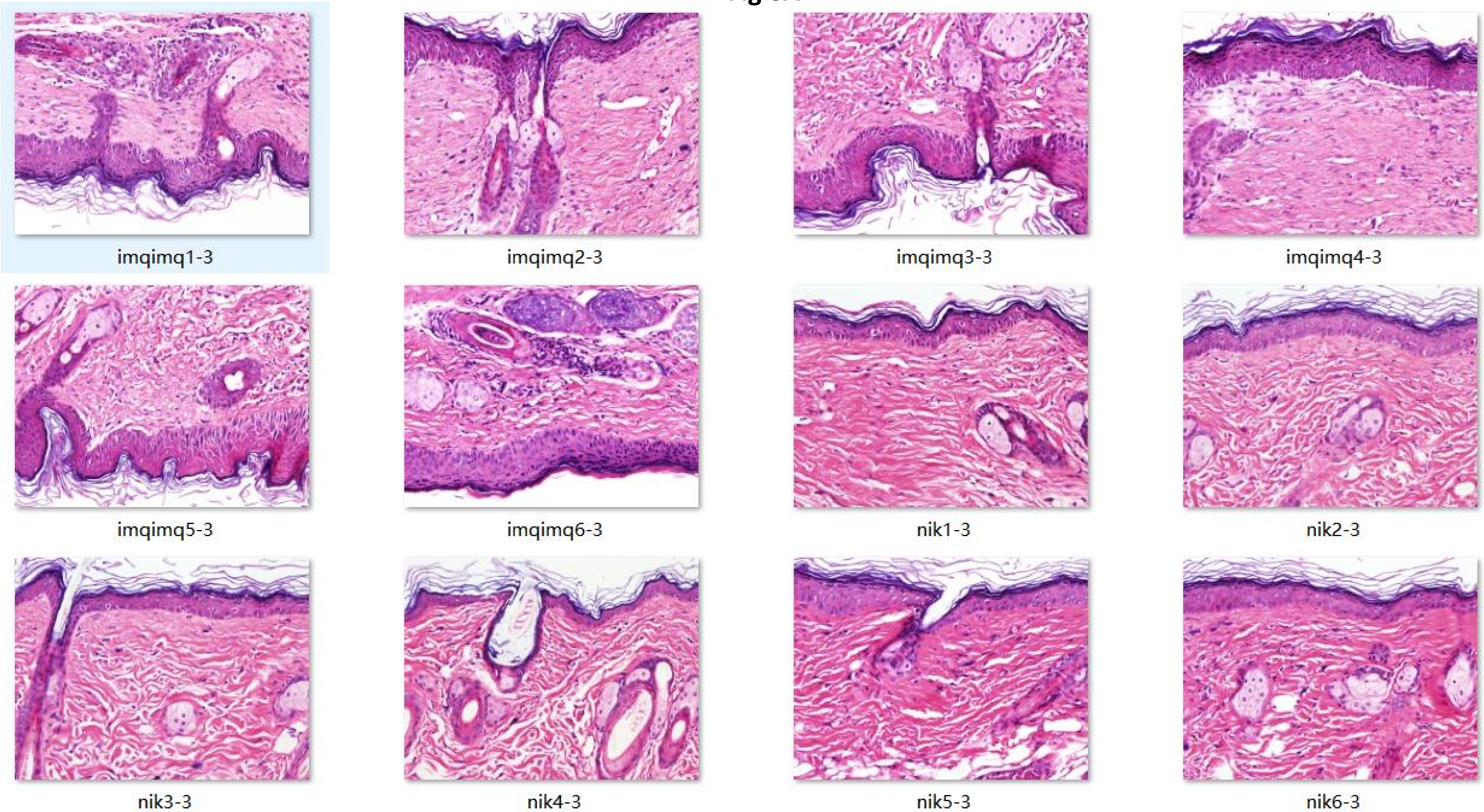
imqimq1-3
imqimq2-3
imqimq3-3
imqimq4-3
imqimq5-3
imqimq6-3
nik1-3
nik2-3
nik3-3
nik4-3
nik5-3
nik6-3

Fig. 5 GAPDH

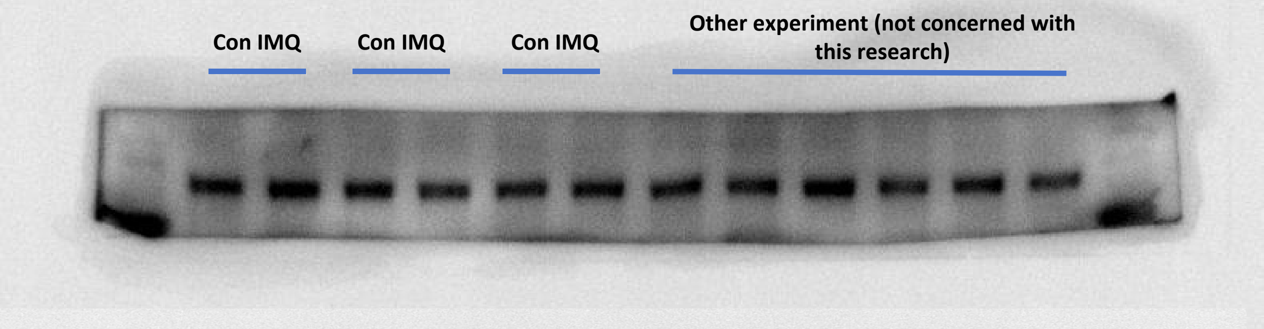

Fig. 5 p-IKKα

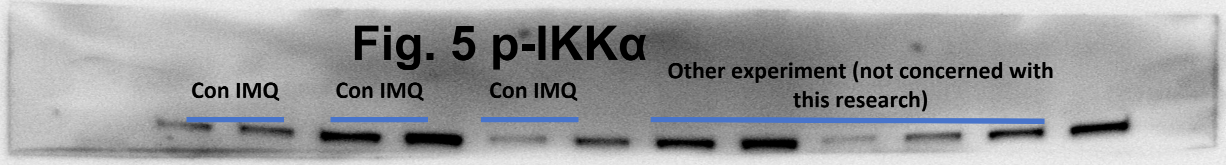

Fig. 5 p-RelB

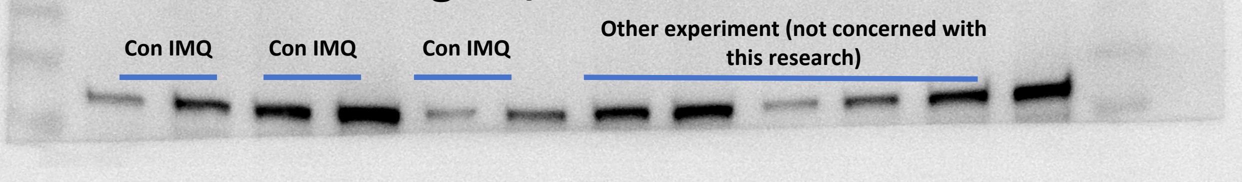

Fig. 5 p-NFkB p65

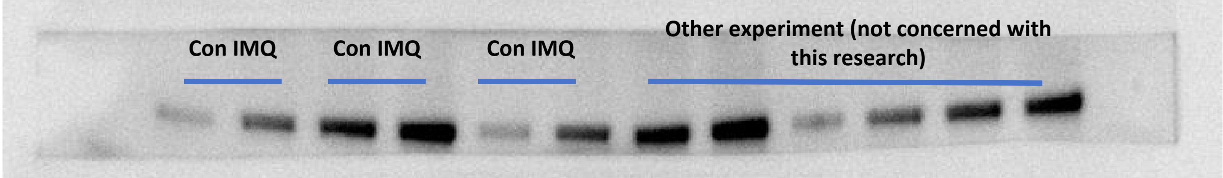

Fig. 5 NFκBp100

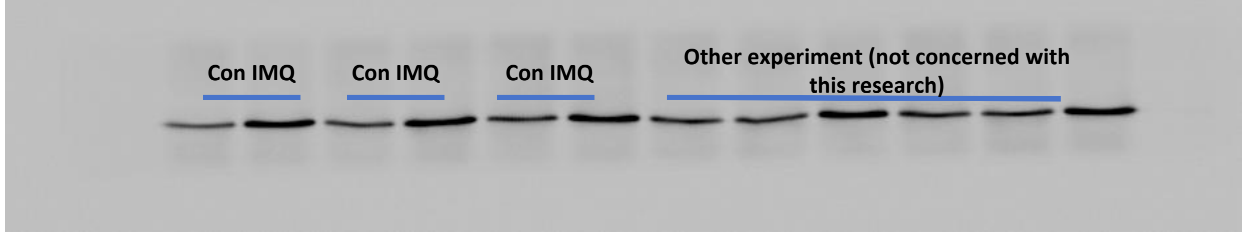

Fig. 5 GAPDH

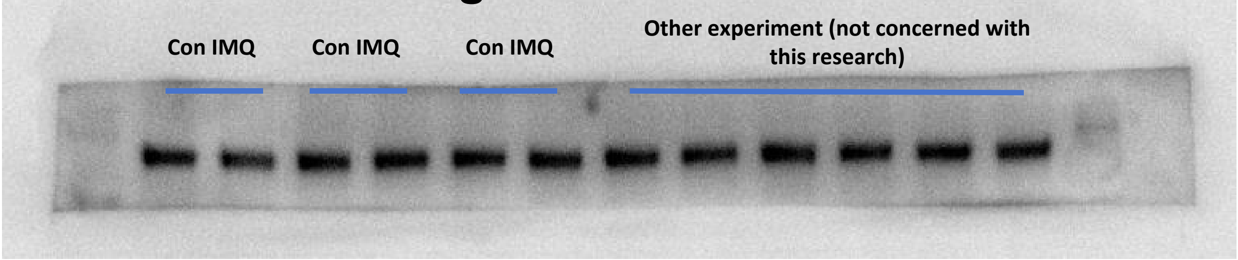

Fig. 5 p-MAPK p38

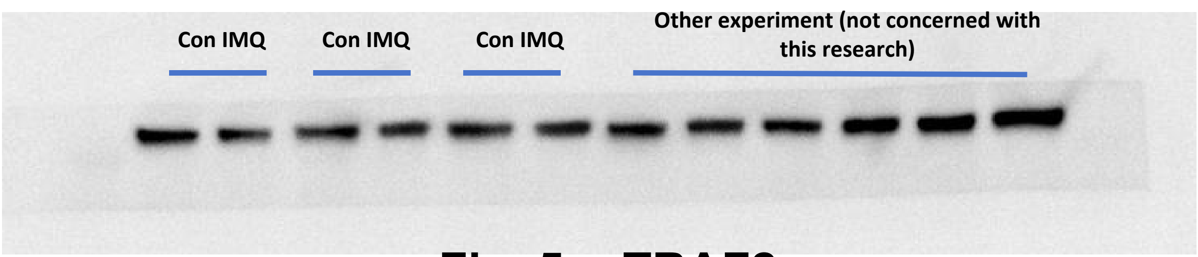

Fig. 5 p-TRAF2

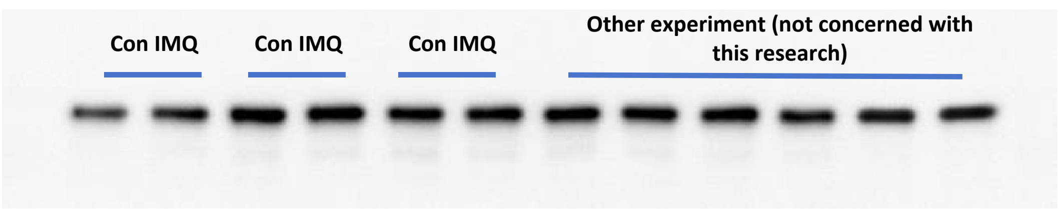

Fig. 5 NFκBp52

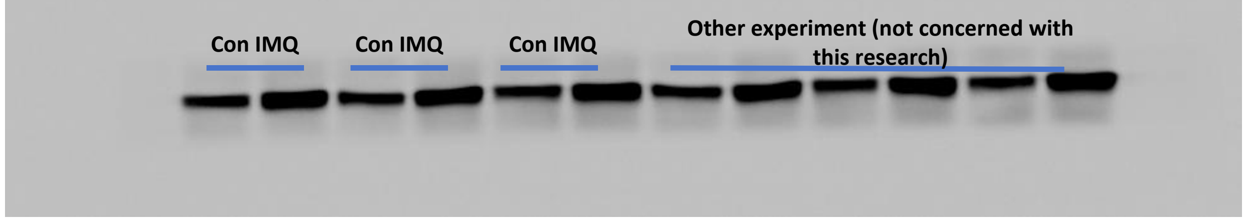

Supplement: Supplementary file 2 [file DataSheet1.pdf]
